# Prognostic Impact of a Simple GDMT Score on Clinical Outcomes and Cardiac Reverse Remodelling in Patients with ADHF: Insights from the JROADHF-Next Registry

**DOI:** 10.1016/j.cjco.2026.06.022

**Published:** 2026-07-20

**Authors:** Ryuichi Matsukawa, Yasushi Mukai, Yudai Fujimoto, Yuya Matsue, Takafumi Sakamoto, Nobuyuki Enzan, Takeshi Tohyama, Shouji Matsushima, Masataka Ikeda, Takeshi Kitai, Keisuke Kida, Takahiro Okumura, Tatsunori Taniguchi, Tomomi Ide, Hiroyuki Tsutsui, Kohtaro Abe

**Affiliations:** a Division of Heart Failure and Carxdiology, Iizuka Hospital, Fukuoka, Japan; b Division of Cardiology, Fukuoka Red Cross Hospital, Fukuoka, Japan; c Department of Cardiovascular Biology and Medicine, Juntendo University Graduate School of Medicine, Tokyo, Japan; d Department of Cardiovascular Medicine, Faculty of Medical Sciences, Kyushu University, Fukuoka, Japan; e Division of Cardiovascular Medicine, Research Institute of Angiocardiology, Faculty of Medical Sciences, Kyushu University, Fukuoka, Japan; f Cardiovascular Disease Initiative, Broad Institute, Cambridge, Massachusetts, USA; g International University of Health and Welfare, Okawa, Japan; h Centre for Clinical and Translational Research, Kyushu University Hospital, Fukuoka, Japan; i Institute for Medical Engineering and Science, Massachusetts Institute of Technology, Cambridge, Massachusetts, USA; j Department of Cardiovascular Medicine, National Cerebral and Cardiovascular Centre, Osaka, Japan; k Department of Pharmacology, St. Marianna University School of Medicine, Kanagawa, Japan; l Department of Cardiology, Nagoya University Graduate School of Medicine, Nagoya, Japan; m Department of Cardiovascular Medicine, Osaka University Graduate School of Medicine, Osaka, Japan

**Keywords:** heart failure, guideline-directed medical therapy, simple GDMT score, prognosis, cardiac reverse remodelling

## Abstract

**Background:**

Reported results indicate that implementation rates of guideline-directed medical therapy (GDMT) are associated with improved prognosis and cardiac function in heart failure (HF) patients. This study aimed to investigate the relationship between what we term a “simple GDMT score” calculated at discharge and subsequent cardiac reverse remodelling and long-term outcomes in patients hospitalized for acute decompensated HF, using data from the **J**apanese **R**egistry **o**f **A**cute **D**ecompensated **H**eart **F**ailure (JROADHF-NEXT) prospective registry.

**Methods:**

We analyzed 2313 hospitalized patients with HFrEF or HFmrEF, stratifying them into high-score (≥ 5 points) and low-score (< 5 points) groups based on their discharge GDMT score.

**Results:**

The high-score group demonstrated a significantly lower risk of the composite endpoint of cardiovascular death or HF rehospitalization (hazard ratio = 0.64, 95% confidence interval: 0.55-0.76), an association that remained significant following multivariable adjustment (adjusted model: hazard ratio = 0.70, 95% confidence interval: 0.56-0.88). Furthermore, the high-score group exhibited significantly greater improvements in echocardiographic indices, including absolute changes in left ventricular ejection fraction (*P* < 0.001), left ventricular end-diastolic volume index (*P* < 0.001), and left ventricular end-systolic volume index (*P* < 0.001), and showed significant reductions in brain natriuretic peptide (*P* = 0.005) and N-terminal pro brain natriuretic peptide levels (*P* < 0.001) at 1-year follow-up assessment.

**Conclusions:**

A higher simple GDMT score at discharge is independently associated with better 2-year clinical outcomes and favourable cardiac reverse remodelling in this cohort of HFrEF/HFmrEF patients hospitalized for acute decompensated HF. These findings suggest that a simple assessment of GDMT at discharge may serve as a practical benchmark of discharge medical optimization in routine clinical care.

## Central Illustration

**Figure:**
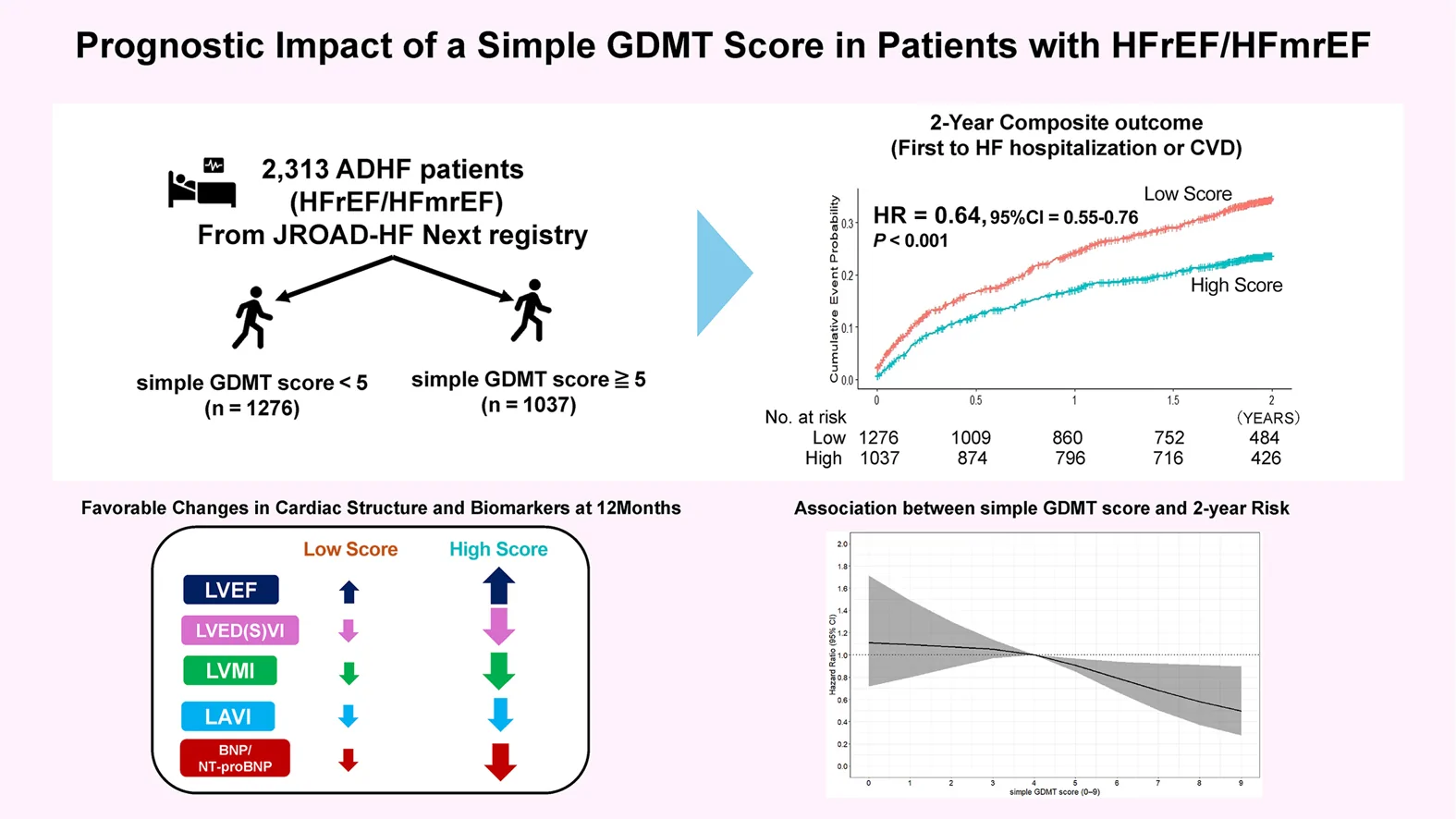

## Résumé

**Contexte:**

Il a été rapporté que des taux plus élevés de mise en œuvre du traitement médical conforme aux lignes directrices (GDMT) sont associés à une amélioration du pronostic et de la fonction cardiaque chez les patients atteints d’insuffisance cardiaque (IC). Cette étude visait à examiner la relation entre un score GDMT simple, calculé au congé, et le remodelage cardiaque inverse ultérieur ainsi que les résultats cliniques à long terme chez les patients hospitalisés pour une IC aiguë décompensée (ICAD), à partir des données du registre prospectif JROADHF-NEXT (Japanese Registry of Acute Decompensated Heart Failure-NEXT).

**Méthodologie:**

Nous avons analysé 2 313 patients hospitalisés atteints d’IC avec fraction d’éjection réduite (ICFEr) ou d’IC avec fraction d’éjection légèrement réduite (ICFElr), en les répartissant en deux groupes : un groupe à score élevé (≥ 5 points) et un groupe à score faible (< 5 points), en fonction de leur score GDMT à la sortie de l’hôpital.

**Résultats:**

Le groupe à score élevé a présenté un risque significativement plus faible de survenue du critère d’évaluation composite associant décès cardiovasculaire ou réhospitalisation pour IC (rapport des risques instantanés [RRI] = 0,64; intervalle de confiance [IC] à 95 % : 0,55–0,76), une association qui est restée significative après ajustement multivarié (modèle ajusté : RRI = 0,70; IC à 95 % : 0,56–0,88). De plus, le groupe à score élevé a présenté des améliorations significativement plus marquées des paramètres échocardiographiques, notamment les variations absolues de la fraction d’éjection ventriculaire gauche (*p* < 0,001), de l’indice de volume télédiastolique ventriculaire gauche (*p* < 0,001) et de l’indice de volume télésystolique ventriculaire gauche (*p* < 0,001), ainsi que des baisses significatives des taux de peptide natriurétique de type B (*p* = 0,005) et propeptide natriurétique de type B N-terminal (*p* < 0,001) lors du suivi à un an.

**Conclusions:**

Dans cette cohorte de patients atteints d’ICFEr ou d’ICFElr hospitalisés pour une ICAD, un score GDMT simple plus élevé à la sortie de l’hôpital est indépendamment associé à de meilleurs résultats cliniques à 2 ans et à un remodelage cardiaque inverse favorable. Ces résultats suggèrent qu’une évaluation simple du score GDMT à la sortie de l’hôpital pourrait servir de référence pratique pour l’optimisation médicale à la sortie dans la pratique clinique courante.

Heart failure (HF) is an increasingly prevalent condition in aging societies, posing a major public health challenge due to its high mortality incidence and associated healthcare costs. The majority of patients with HF carry a poor prognosis, and they impose a substantial economic burden on healthcare systems.^1^^,^^2^ Guideline-directed medical therapy (GDMT) for HF, particularly HF with reduced ejection fraction (HFrEF), has advanced considerably in recent years.^3^^,^^4^ Particularly in those hospitalized for decompensated HF, appropriate implementation of GDMT during the index hospitalization has been shown to reduce mortality and rehospitalization rates^5^ and is strongly recommended in national and international HF guidelines.^6^, ^7^, ^8^

In HFrEF and HF with mildly reduced ejection fraction (HFmrEF), combination therapy, not monotherapy, is emphasized, with quadruple therapy now being the standard recommendation.^9^, ^10^, ^11^, ^12^ Although the primary evidence for this framework was established initially within the HFrEF phenotype, a wealth of contemporary data from pivotal clinical trials, post-hoc subgroup analyses, and large-scale meta-analyses standardly supports the extension of these benefits to the HFmrEF population.^13^ Specifically, individual patient-level data have substantiated that beta-blockers significantly reduce mortality in patients with a mildly reduced left ventricular ejection fraction (LVEF),^14^ and the relative risk reductions driven by sacubitril/valsartan have a robust span across the LVEF spectrum of up to 55%-60%, thereby explicitly covering the HFmrEF range.^15^ In addition, mineralocorticoid receptor antagonists have demonstrated significant efficacy in attenuating HF hospitalizations at the lower end of the non-reduced LVEF spectrum,^16^ and landmark clinical trials evaluating sodium-glucose cotransporter 2 (SGLT2) inhibitors have definitively confirmed major reductions in cardiovascular events within the HFmrEF population.^17^ Because patients with an LVEF < 50% exhibit overlapping mechanisms of adverse ventricular remodelling and shared therapeutic responses to neurohormonal and metabolic blockade, evaluating the comprehensive implementation intensity of GDMT across the entire non-HFpEF spectrum is highly relevant to contemporary clinical practice. Reflecting this relevance, current guidelines recommend these therapies (class I-IIa) for patients with HFmrEF. Consequently, we defined the inclusion criteria for this analysis as being in the non-HFpEF population. However, real-world implementation of GDMT remains suboptimal due to various factors, including comorbidities, frailty, and clinical condition at admission. Many patients who are eligible for GDMT do not receive it at adequate doses or in appropriate combinations, highlighting a significant gap in care that is a concern not only in Japan but globally.^18^, ^19^, ^20^, ^21^

Previously, we developed what we term a “simple GDMT score” incorporating not only whether a patient is prescribed specific GDMT, but also for the dosage of each therapy. We demonstrated that a higher score at discharge was associated with improved 1-year outcomes in patients hospitalized for HF.^22^ We also reported that optimizing GDMT during hospitalization, particularly among HF readmitted patients, could improve the GDMT score and lead to a better 1-year prognosis.^23^ Recently, the multicentre, retrospective observational study showed that a higher simple GDMT score is associated with a lower incidence of outpatient worsening HF and an improved prognosis.^24^ Several GDMT components, including renin-angiotensin system inhibitors,^25^^,^^26^ beta- blockers,^27^ SGLT2) inhibitors,^28^ and mineralocorticoid receptor antagonists (MRAs)^29^ have each been shown to promote reverse remodelling. However, little is known about the cumulative effect of combination GDMT on subsequent cardiac structural and functional changes.

In this study, we aimed to investigate the association between the simple GDMT score at discharge with long-term clinical outcomes and reverse cardiac remodelling using data from the **J**apanese **R**egistry **o**f **A**cute **D**ecompensated **H**eart **F**ailure (JROADHF-NEXT), a large-scale, multicentre, prospective, observational study conducted in Japan.^30^

## Material and Methods

### Study design and population

The JROADHF-NEXT registry is a nationwide, multicentre, prospective cohort study of hospitalized patients with acute decompensated HF (ADHF) in Japan.^30^ The inclusion criteria were as follows: (i) hospitalization due to ADHF requiring active management; (ii) a diagnosis of HF based on the Japanese Circulation Society 2011 Guidelines for the Treatment of Acute Heart Failure; (iii) age ≥ 20 years; (iv) brain natriuretic peptide (BNP) ≥ 100 pg/mL or N-terminal pro BNP (NT-proBNP) ≥ 300 pg/mL at admission; and (v) written informed consent.

Exclusion criteria included the following: (i) heart transplantation; (ii) acute coronary syndrome requiring emergent revascularization; (iii) in-hospital death; and (iv) patients deemed unsuitable by the attending physician. Patient enrollment occurred between February 2019 and June 2021. In addition, for the present subanalysis, patients without echocardiographic data, HFpEF patients, and patients receiving maintenance dialysis were excluded from the final analytic cohort, as shown in the study flowchart (Fig. 1). Patients were followed postdischarge according to the predefined, structured schedule of the JROADHF-NEXT registry. To ensure longitudinal data consistency and minimize selection bias, the 1-year follow-up clinical assessments—including repeat echocardiography and biomarker measurements—were mandated strictly according to a prespecified protocol schedule (detailed in Online Table 3 of the registry's baseline report^30^) and performed standardly under stable outpatient clinical conditions at 1 year after discharge, rather than being driven by unstructured clinical judgment or treating physicians' discretion. Although clinical management during the hospital stay remained at the discretion of the treating physicians, the collection of longitudinal data was strictly standardized per the registry protocol to provide a self-contained and consistent evaluation of patient outcomes. Further details of the registry design have been described previously.^30^ Of note, baseline echocardiographic data were uniformly obtained after hemodynamic stabilization and before hospital discharge. To ensure rigorous event tracking, the JROADHF-NEXT framework uniquely linked the prospective clinical registry with the institutional Diagnosis Procedure Combination (DPC) electronic administrative claim database. All clinical outcomes, including heart failure rehospitalization and cardiovascular death, were tracked and adjudicated by experienced site cardiologists based on strict, protocol-specified definitions (cardiovascular death was defined as death due to myocardial infarction, HF, ventricular tachyarrhythmia, stroke, pulmonary embolism, or other validated aortic/vascular diseases). Information on comorbidities was collected at the time of discharge and partially collected at the 1-year follow-up assessment. The median follow-up period was 2.0 (1.6-2.2) years.

**Figure 1:**
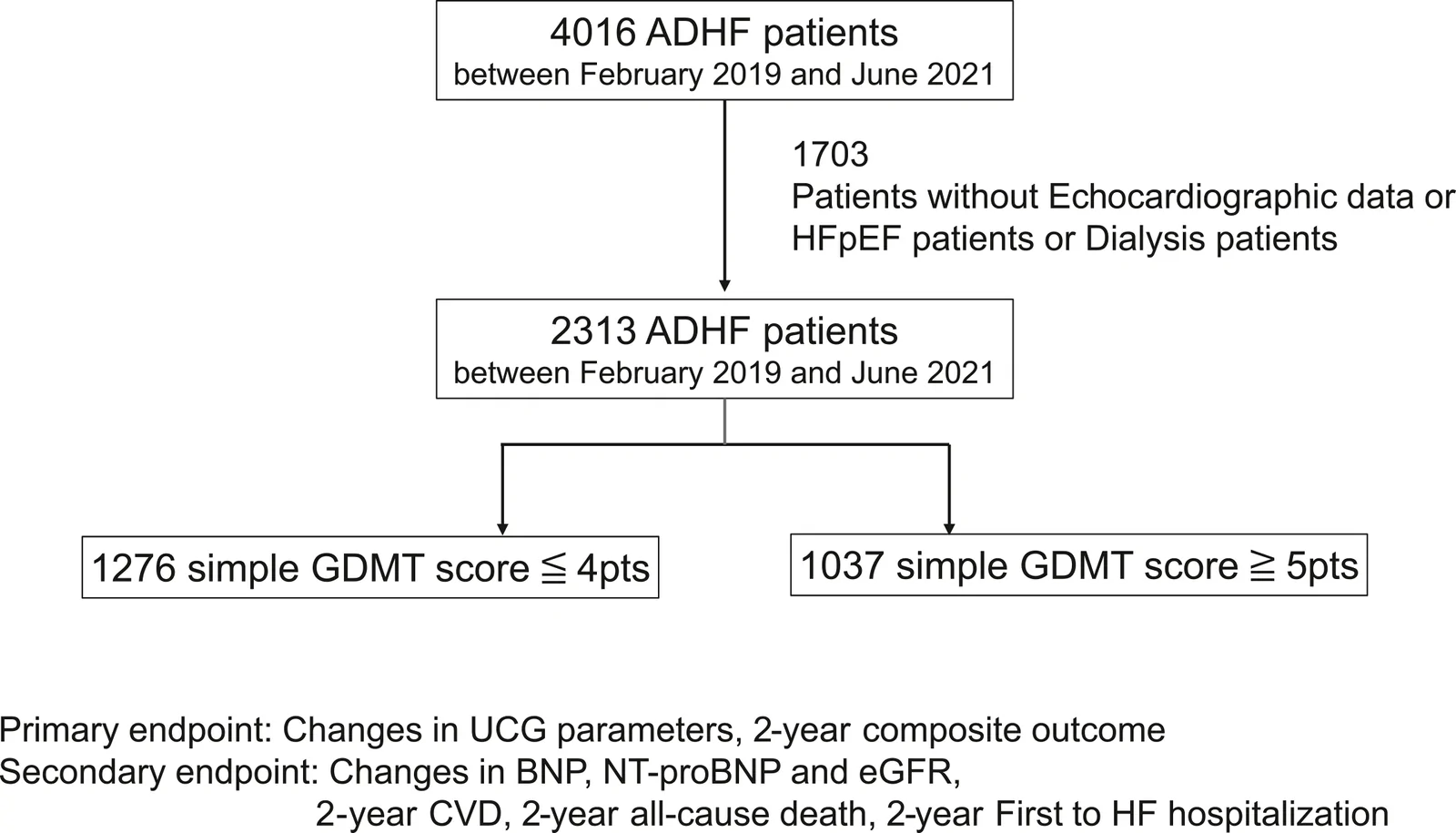
Study design,definition, and regime of simple guideline-directed medical therapy (GDMT) score. ADHF, acute decompensated heart failure; BNP, brain natriuretic peptide; CVD, cardiovascular death; eGFR, estimated glomerular filtration rate; HFpEF, heart failure with preserved ejection fraction; NT-pro BNP, N-terminal pro BNP; pts, points; UCG,

**Table 3:** Multivariable Cox proportional hazards analysis

Association with clinical outcomes	Unadjusted model			Adjusted model		
	HR	95% CI	*P*	HR	95% CI	*P*
Composite outcome	0.64	0.55–0.76	< 0.001	0.70	0.56–0.88	0.002
HF hospitalization	0.71	0.60–0.84	< 0.001	0.77	0.61–0.98	0.03
CV death	0.38	0.28–0.53	< 0.001	0.56	0.37–0.84	0.005
All-cause death	0.46	0.37–0.58	< 0.001	0.66	0.50–0.89	0.006

The adjusted model was adjusted for validated Get With The Guideline-Heart Failure (GWTG-HF) risk score, estimated glomerular filtration rate, log transformed brain natriuretic peptide at discharge, New York Heart Association functional class at discharge, left ventricular ejection fraction at discharge, anemia, and and prior heart failure (HF) hospitalization.

CI, confidence interval; CV, cardiovascular; HR, hazard ratio.

### Calculation of the simple GDMT score

As previously described,^22^ we calculated the simple GDMT score at discharge based on the prescription and dosing of 4 key drug classes. The clinical and medication data utilized for this scoring were derived directly from the official hospital discharge prescription records. These data were systematically extracted from institutional electronic medical records and entered into a standardized centralized electronic data capture (EDC) system by the site investigators at each participating centre. As noted in the Limitations section, detailed longitudinal data regarding subsequent postdischarge outpatient prescription adjustments or individual patient medication adherence over the 2-year follow-up period were not available in the registry protocol. Therefore, this score reflects the baseline guideline implementation status optimized at the time of hospital discharge: renin–angiotensin system (RAS) inhibitors, beta-blockers (BBs), mineralocorticoid receptor antagonists (MRAs), and SGLT2 inhibitors. RAS inhibitors and BBs were scored as follows: 0 if not prescribed, 1 if prescribed at < 50% of the target dose, and 2 if prescribed at 50%-100% of the target dose. Angiotensin receptor neprilysin inhibitor (ARNIs) were scored as 3 points, regardless of dose. MRAs and SGLT2 inhibitors were given a score of 0 if not prescribed and 2 if prescribed, regardless of dose (Fig. 1). The total score ranged from 0 to 9. Regarding drugs not directly indicated for HF, the ratio of the dose to the maximum approved dose in Japan was calculated (see Supplemental Table S1).

### Study design and outcomes

An overview of the study design is shown in Figure 1. Based on the cutoff used in a prior study,^22^ patients were stratified into 2 groups as follows: high GDMT score (≥ 5 points) and low GDMT score (< 5 points). The receiver operating characteristics curve of the association between the simple GDMT score and the composite outcome is shown in Supplemental Figure S1 (cutoff, 4.5; sensitivity, 0.633; specificity, 0.480; AUC (area under curve), 0.564).

First, long-term outcomes were assessed. We compared the cumulative incidence of 2-year cardiovascular death or HF rehospitalization (composite outcome), cardiovascular death, all-cause death, and HF rehospitalization between the 2 GDMT score groups. Cox proportional hazards models were used to examine the association between the GDMT score group and the composite outcome.

We secondly evaluated changes in echocardiographic parameters between discharge and 1-year follow-up, including LVEF, left ventricular end-diastolic volume index (LVEDVI), LV end-systolic volume index (LVESVI), left atrial volume index (LAVI), left ventricular mass index (LVMI), and E/E′. Absolute change and percent change from baseline were calculated and compared between the groups. Similarly, we compared absolute change and percent changes in biomarkers (log-transformed BNP and log-transformed NT-proBNP) and estimated glomerular filtration rate (eGFR) between discharge and 1-year follow-up assessment. Table 4 shows the number of patients who were followed up with echocardiography at 1 year.

### Statistical analysis

Continuous variables are presented as mean ± standard deviation or median with interquartile range (IQR), as appropriate. No imputation was performed, and analyses were conducted as complete case analyses. Group comparisons were performed using the Student *t* test or the Wilcoxon rank-sum test for continuous variables, and the χ^2^ or Fisher’s exact test for categorical variables. Survival analyses were conducted using Kaplan–Meier methods and compared with log-rank tests. The time to event was defined as the period from discharge of index hospitalization to the event. Cox proportional hazards models were used to estimate hazard ratios (HRs) and 95% confidence intervals (CIs). The proportional hazards assumption was tested using Schoenfeld residuals. Adjustment variables included the validated GWTG-HF (Get-With-The-Guideline-HF) score, eGFR at discharge, log-transformed BNP level at discharge, NYHA (New York Heart Association) functional class at discharge, LVEF at discharge, anemia, and prior HF hospitalization. The validated GWTG-HF score has been described previously.^31^ This validated score was calculated from the original GWTG-HF score,^32^ which is based on data at hospital discharge including systolic blood pressure, blood urea nitrogen (BUN) level, sodium values, age, heart rate, and COPD (chronic obstructive pulmonary disease). This score has been validated as an independent predictor of short- and long-term outcomes across HF phenotypes in Japanese populations. Nutritional status at discharge was assessed using the Geriatric Nutritional Risk Index (GNRI), a validated screening tool for elderly patients.^33^ The GNRI was calculated using the following formula: 14.89 x serum albumin (g/dL) + 41.7 x (present body weight / ideal body weight). GNRI has been established as a reliable predictor of clinical outcomes and mortality in patients with heart failure, reflecting both protein and energy nutritional status. Frailty status was evaluated using the Clinical Frailty Scale (CFS),^34^ a validated 9-point scale ranging from 1 (very fit) to 9 (terminally ill). The CFS reflects the overall degree of frailty based on clinical judgment, taking into account functional capacity, comorbidity burden, and dependence in activities of daily living. Higher scores indicate more advanced frailty.

To explore the nonlinear association between the simple GDMT score and the 2-year risk of the composite outcome (HF rehospitalization or all-cause death), we used restricted cubic spline regression modelling based on a Cox proportional hazards model. The model included the simple GDMT score as a continuous variable modelled using restricted cubic splines with 3 knots, adjusted for GWTG score, log-transformed BNP, NYHA class, LVEF, presence of anemia, and history of prior HF hospitalization.

A 2-sided *P*-value < 0.05 was considered statistically significant. All analyses were performed using R version 4.4.1 (R Foundation for Statistical Computing, Vienna, Austria).

## Ethics

This study was conducted in accordance with the Declaration of Helsinki. The study protocol was approved by the institutional review boards of Kyushu University (No. 2019-023), the International University of Health and Welfare (No. 24-KS-005), and all participating institutions. Written informed consent was obtained from all participants. The authors had full access to the data and take full responsibility for its integrity and analysis.

## Results

### Patient characteristics and discharge status

Of the 4016 patients enrolled, we excluded those with HFpEF (LVEF ≥ 50%) and those receiving maintenance dialysis. The final analytic cohort included 2313 patients with HFrEF (LVEF ≤ 40%) or HFmrEF (LVEF 41% to 49%). Patient characteristics stratified by high/low GDMT score are shown in Table 1. The patient characteristics for HFrEF and HFmrEF are presented in Supplemental Tables S2 and S3. Overall, a high simple GDMT score was associated with younger age, male sex, higher diastolic blood pressure, higher heart rate, higher body mass index, higher Geriatric Nutritional Risk Index (GNRI), and a lower level of frailty as defined by a lower CFS score. Their LVEF at discharge was lower compared to those with a low GDMT score. Regarding underlying etiology, the proportion of cardiomyopathy was higher, whereas the prevalence of valvular heart disease was lower in the high GDMT–score group. In terms of comorbidities, the high GDMT–score group had a lower prevalence of atrial fibrillation but higher rates of diabetes mellitus and sleep apnea syndrome (Table 1).

**Table 1:** Characteristics of groups with low vs high simple guideline-directed medical therapy scores

Characteristic	Low simple GDMT score, (n = 1276)	High simple GDMT score, (n = 1037)	*P*
**Backgrounds**			
Age, Y	72.2 ± 14.0	65.8 ± 14.2	< 0.001
Male	832 (65.2)	774 (74.6)	< 0.001
Prior HF hospitalization	504 (39.5)	378 (36.5)	0.145
NYHA class 3-4 at admission)	1127 (88.3)	935 (90.2)	0.177
**Etiology**			
IHD	353 (27.7)	289 (27.9)	0.926
Cardiomyopathy	388 (30.4)	380 (36.6)	0.002
Hypertensive heart disease	148 (11.6)	146 (14.1)	0.079
Valvular heart disease	162 (12.7)	63 (6.1)	< 0.001
Others	225 (17.6)	159 (15.3)	0.144
**Comorbidities**			
AF	488 (38.2)	350 (33.8)	0.027
DM	379 (29.7)	489 (47.2)	< 0.001
DL	493 (38.6)	435 (41.9)	0.115
COPD	86 (6.7)	59 (5.7)	0.343
SAS	57 (4.5)	91 (8.8)	< 0.001
Cancer	115 (9.0)	75 (7.2)	0.128
**Characteristics at discharge**			
NYHA class 3-4	117 (13.5)	87 (8.4)	< 0.001
Systolic BP, mm Hg	109.2 ± 17.0	107.7 ± 17.0	0.036
Diastolic BP, mm Hg	63 (56, 71)	64 (57, 73)	0.004
Heart rate, bpm	70 (63, 80)	70 (62, 78)	0.237
BMI, kg/m^2^	21.0 (18.7, 23.3)	22.5 (20.0, 25.4)	< 0.001
GNRI	90.5 ± 9.0	94.1 ± 8.3	< 0.001
Clinical Frailty Scale	4 (3, 5)	4 (3, 4)	< 0.001
EF at discharge, %	33.8 ± 9.4	31.8 ± 9.1	< 0.001
**Laboratory data at discharge**			
BNP, pg/mL	463.0 ± 533.0	367.4 ± 402.8	< 0.001
NT-proBNP, pg/mL	2153.0 (979.0, 4880.0)	1390.0 (660.7, 2846.7)	< 0.001
Hemoglobin, g/dL	12.3 ± 2.3	13.3 ± 2.3	< 0.001
BUN, mg/dL	25.0 (18.0, 37.0)	22.0 (17.4, 30.0)	< 0.001
Creatinine, mg/dL	1.16 (0.89, 1.64)	1.11 (0.90, 1.41)	< 0.001
eGFR, mL/min per 1.73 m^2^	43.4 (29.3, 59.2)	48.5 (37.6, 61.8)	< 0.001
Sodium, mEq/L	139.0 (136.0, 141.0)	139.0 (137.0, 141.0)	0.468
Pottasium, mEq/L	4.3 (4.0, 4.7)	4.4 (4.1, 4.7)	0.227
Chloride, mEq/L	102.0 (99.0, 105.0)	103 (100.0, 105.0)	0.220
Albumin, mg/dL	3.5 ± 0.4	3.6 ± 0.4	< 0.001
UA, mg/dL	7.0 ± 2.1	6.7 ± 1.8	< 0.001

Values are n (%), mean ± standard deviation, or median (interquartile range), unless otherwise indicated.

AF, atrial fibrillation; BMI, body mass index; BNP, brain natriuretic peptide; BP, blood pressure; bpm, beats per minute; BUN, blood urea nitrogen; COPD, chronic obstructive pulmonary disease; DL, dyslipidemia; DM, diabetes mellitus; EF, ejection fraction; eGFR, estimated glomerular filtration rate; GNRI, Geriatric Nutritional Risk Index; HF, heart failure; IHD, ischemic heart disease; LVEF, left ventricular ejection fraction; mEq, milliequivalents per liter; NT-proBNP; N-terminal pro-brain natriuretic peptide; NYHA, New York Heart Association; SAS, sleep apnea syndrome; UA, uric acid.

At discharge, the high-score group had shorter hospital stays and significantly higher prescription rates of all classes of GDMT (Table 2). The distribution of simple GDMT scores is shown in Supplemental Figure S2.

**Table 2:** Details at discharge for groups with low vs high simple guideline-directed medical therapy (GDMT) scores

	Low simple GDMT score (n = 1276)	High simple GDMT score (n = 1037)	*P*
**Medication at discharge**			
Hospital stay, d	26.9 ± 24.5	24.5 ± 21.4	0.013
Simple GDMT score, points	2.8 ± 1.1	5.9 ± 1.0	< 0.001
RAS inhibitor	869 (78.0)	990 (97.1)	< 0.001
ARNI	9 (1.1)	79 (11.4)	< 0.001
Beta-blocker	1052 (82.4)	1019 (98.3)	< 0.001
MRA	604 (47.3)	954 (92.0)	< 0.001
SGLT2 inhibitor	88 (6.9)	474 (45.7)	< 0.001
Ivabradine	20 (2.5)	22 (3.2)	0.531
Loop diuretics	1059 (83.0)	881 (85.0)	0.211
Loop diuretics dose, mg	20.0 (10.0, 40.0)	20.0 (10.0, 40.0)	0.150
TLV	436 (34.2)	340 (32.8)	0.507
Pimobendan	177 (13.9)	116 (11.2)	0.059

Values are mean ± standard deviation, n (%), or median (interquartile range).

ARNI, angiotensin receptor neprilysin inhibitor; MRA, mineralocorticoid receptor antagonist; RAS, renin angiotensin system; SGLT2 inhibitor, sodium glucose transporter 2 inhibitor; TLV, tolvaptan.

### Incidence of composite outcome, HF rehospitalization, cardiovascular death, and all-cause death

Kaplan–Meier analysis demonstrated that being in the high GDMT–score group was associated with significantly lower event rates for all endpoints; composite outcome (hazard ratio [HR] = 0.64, 95% CI: 0.55-0.76, *P* < 0.001), first HF rehospitalization after discharge (HR = 0.71, 95% CI: 0.60-0.84, *P* < 0.001), cardiovascular death (HR = 0.38, 95% CI: 0.28-0.53, *P* < 0.001), and all-cause death (HR = 0.46, 95% CI: 0.37–0.58, *P* < 0.001; Fig. 2 and Central Illustration). These results were consistent when analyzed separately for HFrEF and HFmrEF (Supplemental Fig. S3).

**Figure 2:**
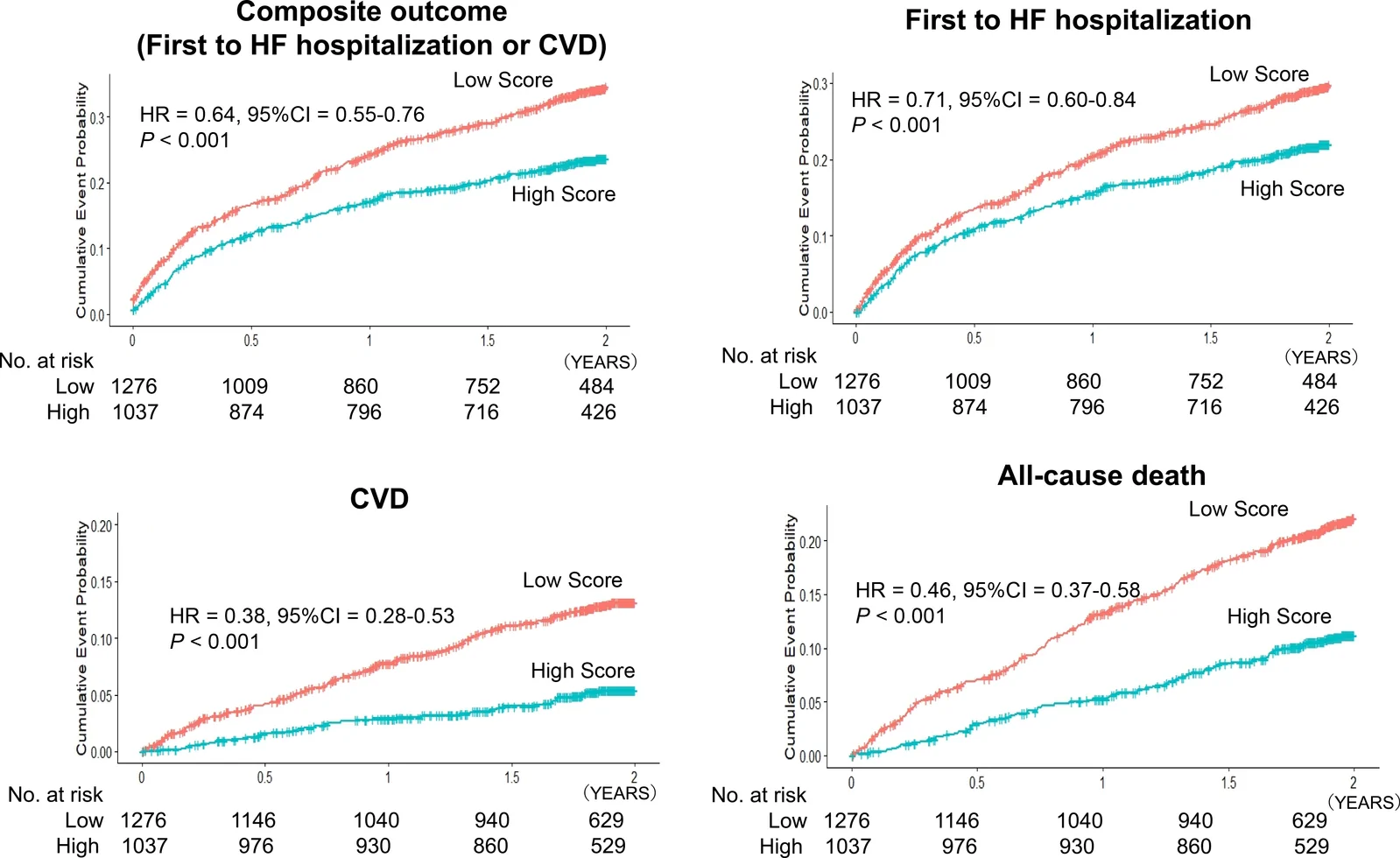
Kaplan–Meier curves for 2-year outcomes— composite outcome (first to heart failure [HF] hospitalization or cardiovascular death [CVD]), first to HF hospitalization, CVD, and all-cause death. CI, confidence interval; HR, hazard ratio.

**Central Illustration:**
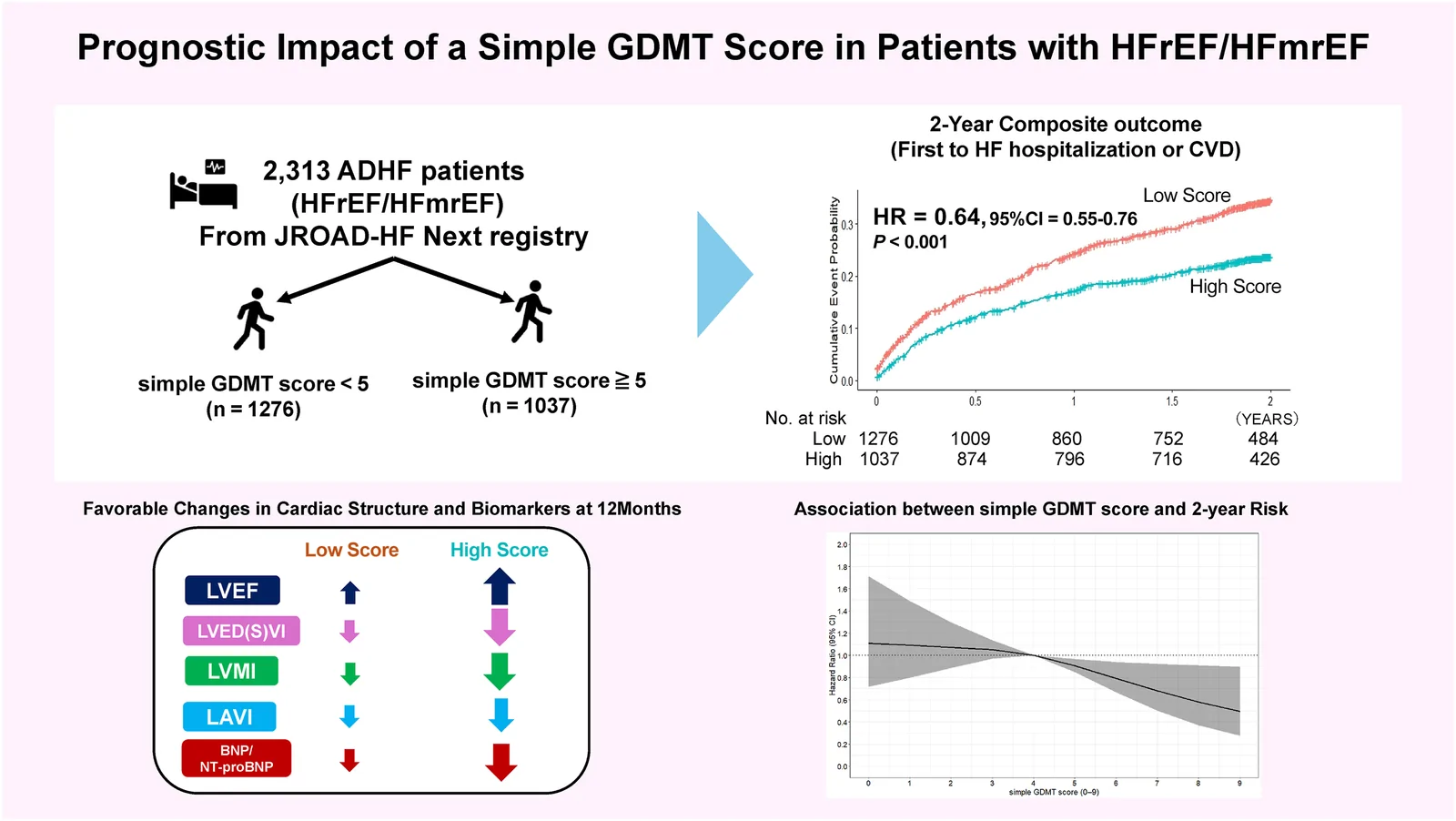
Prognostic impact of a simple guideline-directed medical therapy (GDMT) score in patients with heart failure (HF) with reduced ejection fraction (HFrEF)/HF with mildly reduced ejection fraction (HFmrEF). ADHF, acute decompensated HF; BNP, brain natriuretic peptide; CI, confidence interval; CVD, cardiovascular disease; HR, hazard ratio; JROAD-HF-Next, **J**apanese **R**egistry **o**f **A**cute **D**ecompensated **H**eart **F**ailure; LAVI, left atrial volume index; LVED(S)VI, left ventricular end-diastolic (systolic) volume index; LVEF, left ventricular ejection fraction; LVMI, left ventricular mass index; NT-proBNP, N-terminal pro BNP.

Multivariable Cox proportional hazards analysis confirmed that a high GDMT score remained independently associated with a lower risk of the composite outcome after adjusting for GWTG-HF risk score, log transformed BNP level at discharge, NYHA class at discharge, LVEF at discharge, anemia, and prior HF hospitalization (Table 3). No significant violations were observed. These results were also consistent when analyzed separately for HFrEF and HFmrEF (Supplemental Table S4).

To evaluate the robustness of this association against residual confounding from patient vulnerability, aging, and frailty, we conducted rigorous sensitivity analyses focusing on the primary composite outcome. In Sensitivity Model A, we additionally adjusted for baseline nutritional status (GNRI) and physical frailty (CFS). Strikingly, a high simple GDMT score remained significantly associated with a lower risk of the composite outcome (adjusted HR: 0.760, 95% CI: 0.605-0.955, *P* = 0.018; Supplemental Table S5). In Sensitivity Model B, to satisfy the reviewer's recommendation regarding age while strictly avoiding overadjustment and multicollinearity driven by variable duplication, the composite GWTG-HF risk score was removed from the model and replaced by Age as an independent continuous variable, alongside GNRI and CFS score. Remarkably, even after controlling for these potent physiological drivers, a high simple GDMT score maintained its independent and significant association with a lower risk of the 2-year composite outcome (Sensitivity Model B: adjusted HR: 0.806, 95% CI: 0.656-0.989, *P* = 0.039; Supplemental Table S5).

The restricted cubic spline analysis revealed a nonlinear inverse association between the simple GDMT score and the risk of 2-year composite outcomes (Fig. 3). The hazard ratio decreased gradually as the GDMT score increased, with the risk plateauing at higher score levels. In particular, patients with a score ≥ 5 demonstrated a significantly lower HR compared to those with lower scores, indicating a potential threshold effect.

**Figure 3:**
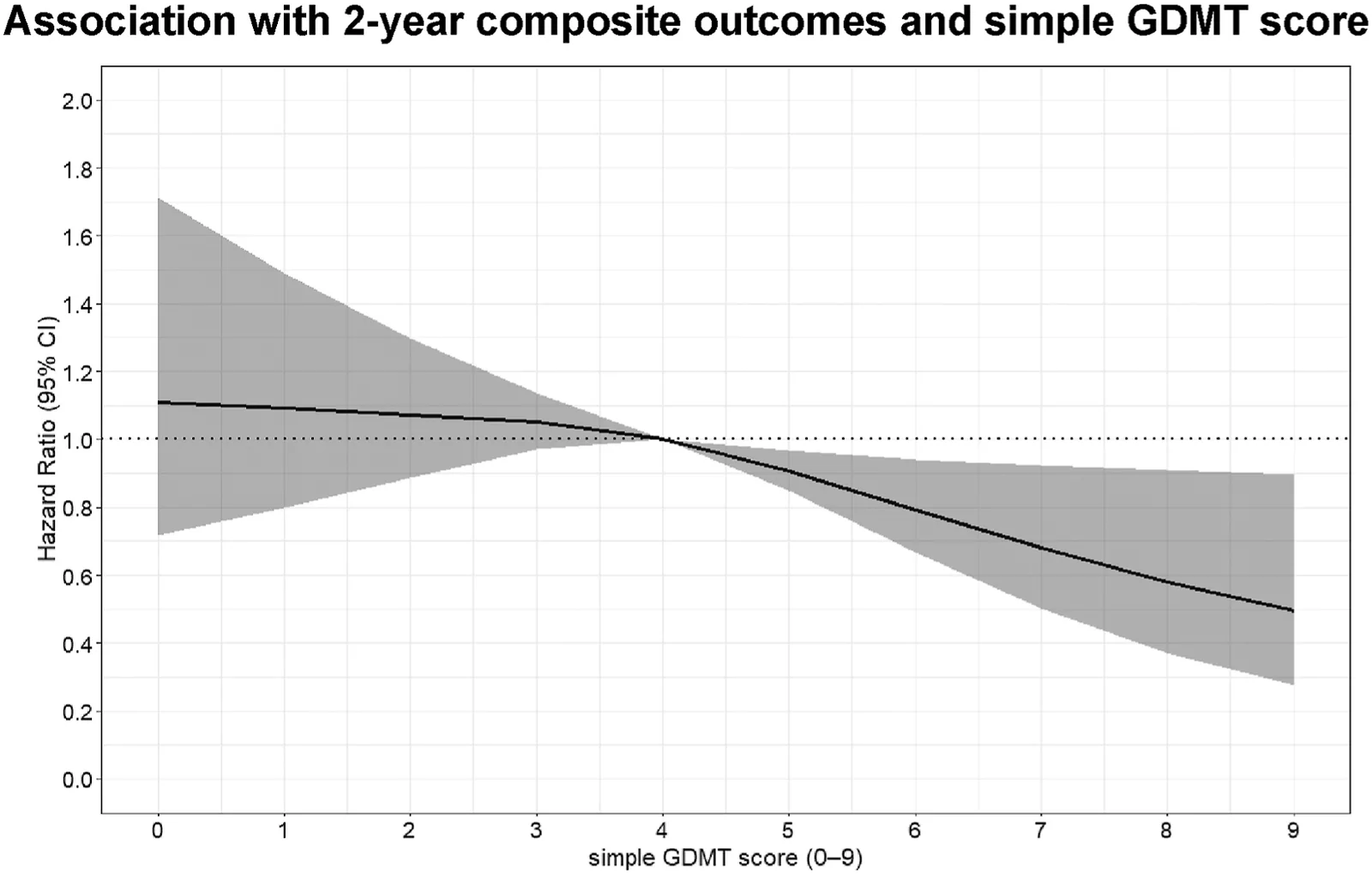
Cubic spline curve. Restricted cubic spline analysis of the association between the simple guideline-directed medical therapy (GDMT) score and 2-year composite outcome (heart failure rehospitalization or all-cause death). The **solid line** represents the hazard ratio (HR), and the **shaded area** represents the 95% confidence interval (CI). The **dotted horizontal line** indicates the reference hazard ratio (1.0).

### Changes in echocardiographic parameters and biomarkers between GDMT score groups

Changes in echocardiographic parameters and biomarkers between discharge and 12 months are comprehensively summarized in Table 4, which standardly details the absolute baseline values, 1-year follow-up values, raw absolute changes (delta), relative percentage changes, and their respective, independent group-comparison *P*-values.

**Table 4:** Changes in echocardiographic and biomarker parameters between discharge and 12 months

Parameter	Group	At discharge	*P*	At 1-Yfollow-up	*P*	Absolute change (Δ)	*P*	% change from baseline	*P*-
**LVEF, %**	LS (n = 1276)	35.0 (27.0–42.0) [n = 1276]	< 0.001	44.0 (33.0–54.0) [n = 851]	0.478	8.0 (0.0–18.0) [n = 851]	< 0.001	24.0 (0.0–58.7) [n = 851]	< 0.001
	HS (n = 1037)	32.0 (25.0–39.0) [n = 1037]		45.0 (32.5–56.0) [n = 791]		12.0 (2.0–22.0) [n = 791]		36.7 (5.1–74.1) [n = 791]	
**LVEDVI, mL/m^2^**	LS (n = 1276)	99.3 (77.2–121.1) [n = 1270]	0.001	80.3 (61.2–105.4) [n = 774]	0.821	–11.3 (–30.4–1.6) [n = 774]	< 0.001	–12.0 (–29.9–1.5) [n = 774]	< 0.001
	HS (n = 1037)	102.3 (82.2–126.4) [n = 1035]		78.1 (60.1–108.5) [n = 748]		–18.6 (–36.6–-1.7) [n = 746]		–18.1 (–35.6–-1.9) [n = 746]	
**LVESVI, mL/m^2^**	LS (n = 1276)	64.0 (44.9–87.7) [n = 1267]	< 0.001	43.2 (26.1–68.4) [n = 774]	0.921	–14.3 (–32.3–0.0) [n = 772]	< 0.001	–23.6 (–51.1–0.0) [n = 772]	< 0.001
	HS (n = 1037)	70.1 (51.4–94.8) [n = 1034]		41.5 (25.9–76.4) [n = 748]		–21.2 (–40.3–-4.5) [n = 746]		–32.1 (–58.4–-6.9) [n = 746]	
**LAVI, mL/m^2^**	LS (n = 1276)	49.2 (38.0–67.7) [n = 837]	0.002	40.3 (27.8–57.6) [n = 544]	0.038	–6.7 (–18.7–4.6) [n = 461]	0.180	–13.3 (–35.8–9.6) [n = 461]	0.162
	HS (n = 1037)	47.5 (35.8–61.2) [n = 696]		37.8 (26.4–55.4) [n = 545]		–8.3 (–18.1–2.4) [n = 455]		–17.1 (–36.2–4.8) [n = 455]	
**LVMI, g/m^2^**	LS (n = 1276)	132.3 (108.0–158.9) [n = 1264]	0.378	113.9 (91.4–143.0) [n = 771]	0.026	–12.9 (–36.7–5.2) [n = 767]	< 0.001	–9.9 (–25.8–4.1) [n = 767]	< 0.001
	HS (n = 1037)	132.9 (112.1–158.2) [n = 1035]		110.4 (90.4–136.2) [n = 743]		–19.8 (–40.6–0.4) [n = 741]		–15.6 (–29.8–0.5) [n = 741]	
**E/E'**	LS (n = 1276)	16.6 (11.4–23.2) [n = 1079]	0.403	13.2 (9.5–19.3) [n = 704]	0.003	–1.7 (–6.8–2.7) [n = 639]	0.025	–11.6 (–37.9–22.4) [n = 639]	0.006
	HS (n = 1037)	16.0 (11.7–22.2) [n = 861]		11.9 (8.9–17.1) [n = 652]		-–2.8 (–7.9–1.3) [n = 581]		–19.7 (–43.1–10.7) [n = 581]	
**BNP, pg/mL**	LS (n = 1276)	305.5 (145.2–579.8) [n = 839]	< 0.001	163.8 (50.6–414.9) [n = 617]	< 0.001	–0.5 (–1.5–0.3) [n = 579]	0.002	–8.3 (–27.3–5.0) [n = 579]	0.003
	HS (n = 1037)	261.0 (127.7–475.6) [n = 740]		108.4 (35.8–293.1) [n = 595]		–0.8 (–1.8–0.1) [n = 575]		–14.7 (–32.4–1.5) [n = 575]	
**NT-proBNP, pg/mL**	LS (n = 1276)	2153.0 (979.0–4880.0) [n = 437]	< 0.001	1370.0 (335.0–4194.0) [n = 385]	< 0.001	–0.5 ± 1.5 [n = 322]	< 0.001	–4.1 (–19.0–5.5) [n = 322]	< 0.001
	HS (n = 1037)	1390.5 (660.8–2846.8) [n = 350]		613.0 (177.0–1754.0) [n = 361]		–1.0 ± 1.4 [n = 284]		–12.8 (–26.0–-0.8) [n = 284]	
**eGFR, mL/min per 1.73 m^2^**	LS (n = 1276)	43.4 (29.3–59.2) [n = 1273]	< 0.001	41.2 (28.1–57.9) [n = 1031]	< 0.001	–2.3 (–10.3–5.6) [n = 1031]	0.695	–5.9 (–23.3–14.2) [n = 1031]	0.748
	HS (n = 1037)	48.5 (37.6–61.8) [n = 1037]		47.3 (34.2–61.5) [n = 931]		–2.5 (–11.1–5.4) [n = 930]		–4.9 (–21.8–13.6) [n = 930]	

BNP, brain natriuretic peptide; eGFR, estimated glomerular filtration rate; HS, high score; LAVI, left atrial volume index; LS, low score; LVEDVI, left ventricular end-diastolic volume index; LVEF, left ventricular ejection fraction; LVESVI, left ventricular end-systolic volume index; LVMI, left ventricular mass index; NT-proBNP, N-terminal pro BNP.

In the overall cohort, the high simple GDMT score group demonstrated highly favourable cardiac reverse remodelling across all structural domains. The raw absolute numerical improvements (delta) in LVEF were significantly greater in the high-score group than in the low-score group (12.0% [2.0%-22.0%] vs 8.0% [0.0%-18.0%], *P* for delta < 0.001]. Highly significant raw absolute reductions (delta) were also achieved in the high-score group for LVEDVI ([–18.6 [–36.6 to –1.7] vs –11.3 [–30.4 to 1.6mL/m^2^], *P* for delta < 0.001), LVESVI (–21.2 [–40.3 to –4.5] vs –14.3 [–32.3 to 0.0] mL/m^2^, *P* for delta < 0.001), and LVMI (–19.8 [–40.6 to 0.4] vs –12.9 [–36.7 to 5.2] g/m^2^, *P* for delta < 0.001]. Relative percentage changes for these parameters demonstrated a consistently superior, mirrored trajectory in the high-score group (*P* for % change < 0.001 for LVEF, LVEDVI, LVESVI, and LVMI; Table 4; Fig. 4).

**Figure 4:**
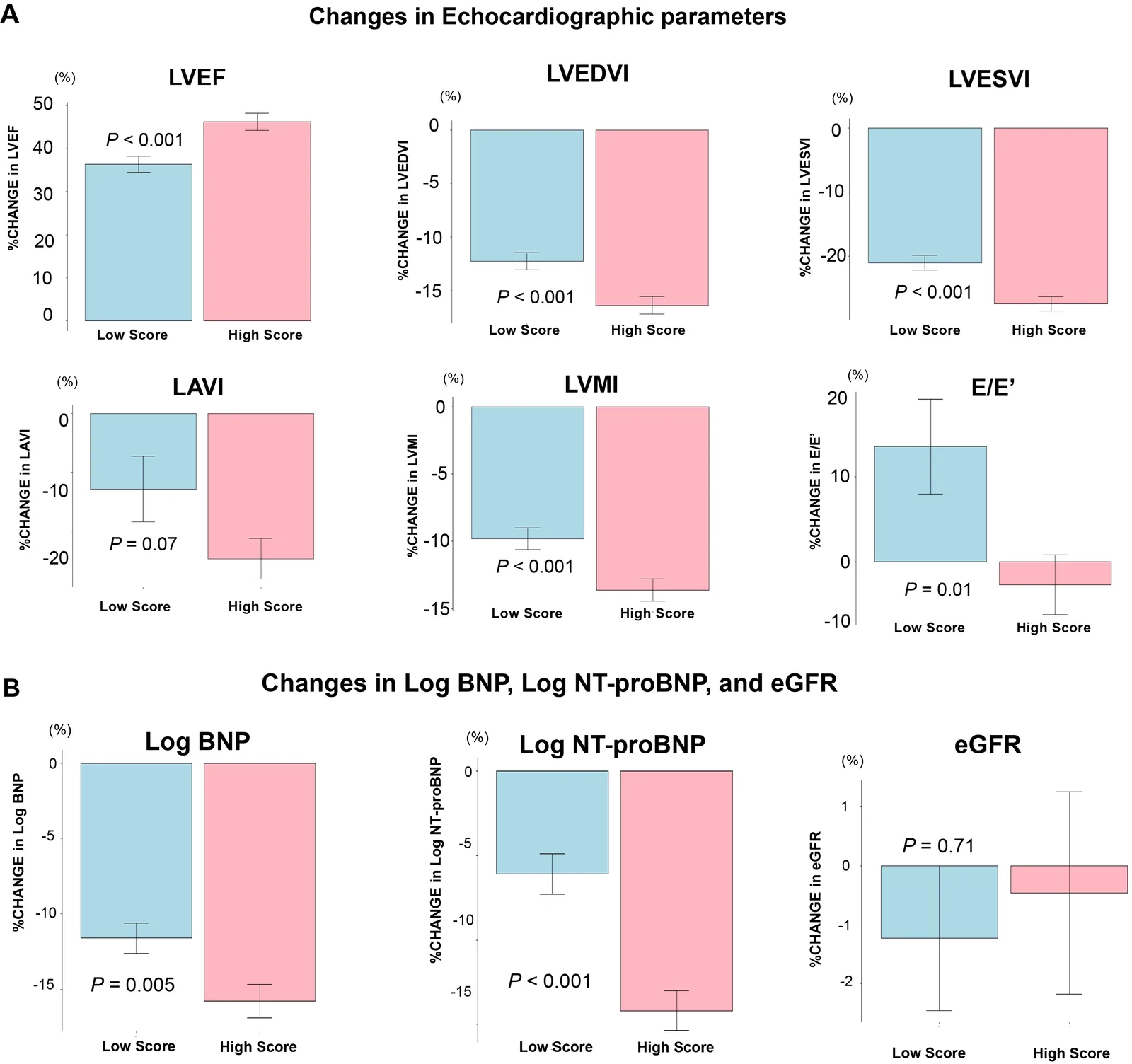
Changes in echocardiographic parameters and biomarkers. (**A**) Percent changes from discharge to 1 year in left ventricular ejection fraction (LVEF), left ventricular end-diastolic volume index (LVEDVI), left ventricular end-systolic volume index (LVESVI), left atrial volume index (LAVI), left ventricular mass index (LVMI), and E/E′. (**B**) Percent changes in log brain natriuretic peptide, log N-termianl-pro brain natriuretic peptide, and estimated glomerular filtration rate. Error bars represent the standard deviation.

Regarding HF biomarkers, the raw reductions in log-transformed BNP level (–0.8 [–1.8 to 0.1] vs –0.5 [–1.5 to 0.3] *P* for delta = 0.002) and log-transformed NT-proBNP level (–1.0 ±1.4 vs –0.5±1.5, *P* for delta < 0.001) were significantly more pronounced in the high-score group (Table 4; Fig. 5). In contrast, no significant difference was observed in the raw absolute change of eGFR between the 2 groups (–2.5 [–11.1 to 5.4] vs –2.3 [–10.3 to 5.6] mL/min per 1.73 m^2^, *P* for delta = 0.695).

**Figure 5:**
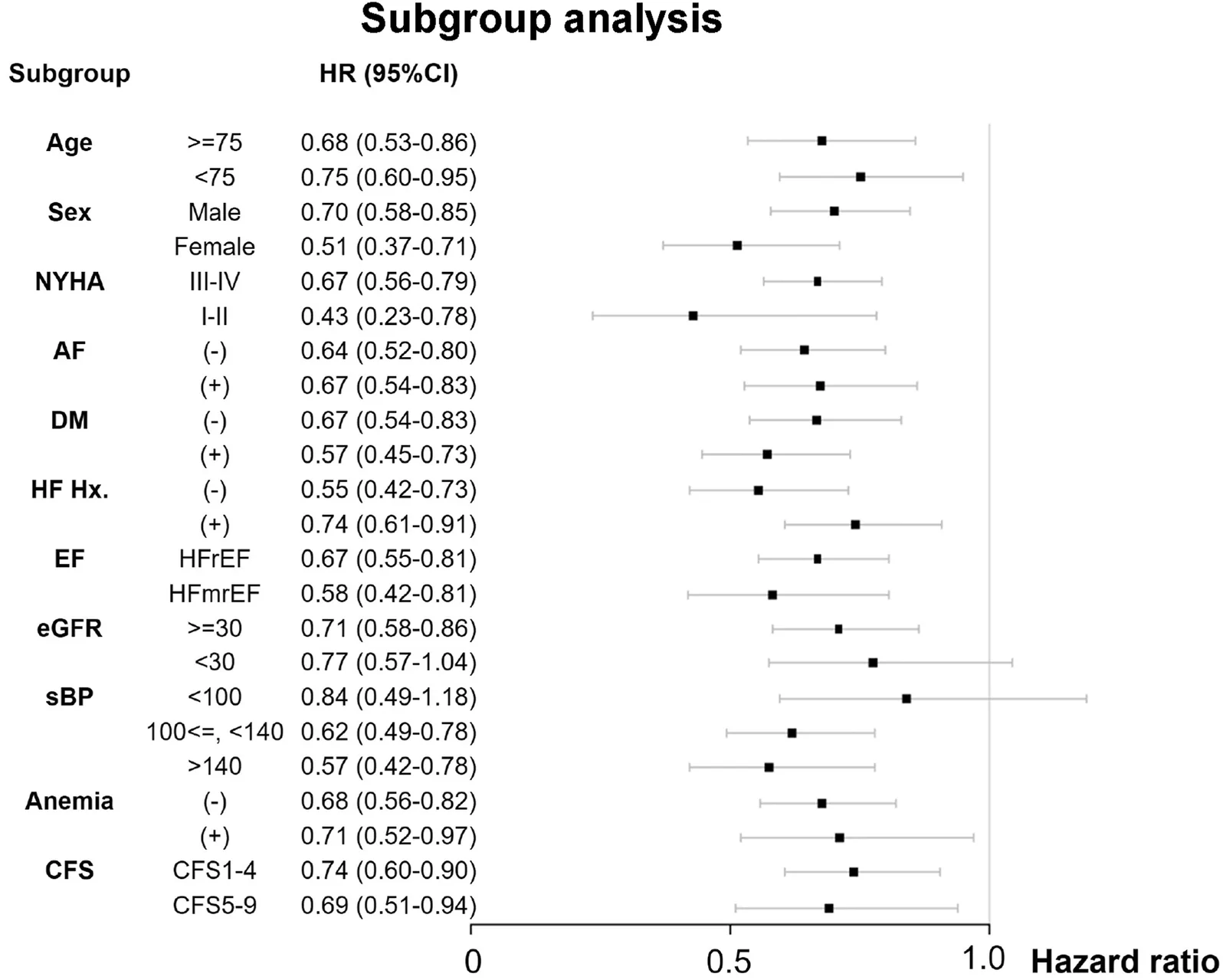
Subgroup analysis for the composite outcome. Forest plot shows the association between the guideline-directed medical therapy score and 2-year composite outcome across subgroups. AF, atrial fibrillation; CFS, Clinical Frailty Scale; CI, confidence interval; DM, diabetes mellitus; EF, ejection fraction; eGFR, estimated glomerular filtration rate; HF Hx, history of heart failure; HFmrEF, heart failure with mildly reduced ejection fraction; HFrEF, heart failure with reduced ejection fraction; HR, hazard ratio; NYHA; New York Heart Association, sBP, systolic blood pressure.

Subgroup analyses stratified by HF phenotype further substantiated the consistent therapeutic efficacy of optimized discharge GDMT across different left ventricular functions. In the HFrEF cohort (LVEF ≤ 40%), patients in the high-score group exhibited significantly greater raw absolute improvements (delta) in LVEF (13.0% [3.0%-25.0%] vs 10.0% [1.0%-21.0%], *P* for delta = 0.012), LVEDVI (–21.5 vs –16.2 mL/m^2^, *P* for delta = 0.008), LVESVI (–25.0 vs –20.1 mL/m^2^, *P* for delta = 0.003), and LVMI (–22.3 vs –14.7 g/m^2^, *P* for delta = 0.012), along with a profound raw reduction in log-transformed NT-proBNP (–1.1 ± 1.4 vs –0.6 ± 1.5, *P* for delta < 0.001; Supplemental Table S6).

Strikingly, the therapeutic benefits extended robustly to the HFmrEF cohort (LVEF 41%-49%), wherein the high-score group similarly achieved significantly greater raw absolute improvements (delta) in LVEF (7.9% ± 11.2% vs 5.2% ± 11.1%, *P* for delta = 0.014), LVEDVI (–11.1 [–24.6 to 1.6] vs –4.1 [–19.1 to 4.8] mL/m^2^, *p* for delta = 0.011), and LVESVI (–11.4 [–21.9 to 0.1] vs –6.8 [–19.5 to 2.8] mL/m^2^, *P* for delta = 0.027), as well as a significant raw decrease in log-transformed BNP (–0.4 [–1.2 to 0.2] vs –0.2 [–0.9 to 0.5], *P* for delta = 0.013; Supplemental Table S7). Longitudinal relative percentage changes within each phenotype are visually represented in Supplemental Figures S4-S6. These multilayered analyses collectively indicate that the clinical and structural benefits driven by discharge GDMT optimization are independent of the baseline HF phenotype.

### Subgroup analysis

Subgroup analyses consistently showed a trend toward better outcomes in the high GDMT–score group across various categories, including age, sex, and the presence or absence of major comorbidities (Fig. 5).

## Discussion

In this multicentre prospective registry study of patients hospitalized with ADHF, we evaluated the utility of the simple GDMT score, a measure that captures not only whether each key HF medication is prescribed but also the extent of dose optimization. Our findings demonstrated that a higher simple GDMT score at discharge was significantly associated with greater improvement in cardiac function (ie, reverse remodelling), greater reduction in biomarker levels, and better 2-year clinical outcomes. In the present study, we included patients with both HFrEF and HFmrEF (LVEF < 50%). This decision was informed by recent evidence indicating that the benefits of foundational GDMT, including ARNI and beta-blockers, extend to the HFmrEF population. Our findings reinforce the clinical importance of implementing comprehensive GDMT in patients across the non-HFpEF spectrum to promote cardiac reverse remodelling and improve long-term outcomes.

We conducted a detailed analysis of echocardiographic and biomarker changes 1 year after discharge. Although the reverse remodelling effects of individual HF medications, such as RAS inhibitors, beta-blockers, MRAs, and SGLT2 inhibitors have been well established in previous studies,^22^, ^23^, ^24^, ^25^, ^26^ data on the effect of combination therapy on reverse remodelling have been limited. Our study is among the first to show that a higher cumulative GDMT implementation, as quantified by the simple GDMT score, correlates with more pronounced reverse remodelling.

Numerous studies and guidelines have underscored the association between GDMT implementation rates and reduced rates of rehospitalization and mortality.^35^^,^^36^ A prior study from our group demonstrated that a higher simple GDMT score at discharge predicted improved 1-year prognosis in patients with ADHF.^22^ Our present results are consistent with and expand upon these findings, indicating that the benefits of GDMT persist over a longer term (2 years), and they may be mediated through favourable cardiac remodelling. In addition, a significant reduction in BNP/NT-proBNP levels was observed in this study. In the Safety, Tolerability and Efficacy of Up-Titration of Guideline-Directed Medical Therapies for Acute Heart Failure (STRONG-HF) trial, early implementation of combination therapy was also associated with a significantly lower NT-proBNP level at 90 days.^5^ In our study, patients with a higher simple GDMT score showed a sustained reduction in BNP/NT-proBNP levels at 1 year, indicating a longer-term biomarker improvement compared to that in the STRONG-HF trial. This finding underscores the potential of combination therapy for not only symptomatic control but also long-term structural and prognostic benefit.

Another key finding of the present study is that the beneficial impact of a high simple GDMT score on structural reverse remodelling was observed consistently across the entire spectrum of heart failure with an LVEF < 50%. We demonstrated that optimized contemporary GDMT significantly improves LVEF, LVEDVI, and LVESVI in both HFrEF and HFmrEF phenotypes, consistent with traditional heart failure trials. Intriguingly, we observed a distinct divergence in biomarker trajectories between the subgroups; the high-score group exhibited a significant reduction in NT-proBNP but not BNP levels within the HFrEF cohort, whereas the opposite trend was observed in the HFmrEF cohort. This phenomenon can be explained pathophysiologically by the higher implementation rate of ARNIs in the high-score HFrEF group. Because ARNI standardly inhibits neprilysin-mediated degradation of BNP, plasma BNP levels can remain paradoxically elevated or show a blunted decline even during successful cardiac reverse remodelling. Conversely, NT-proBNP is not metabolized by neprilysin, thereby serving as a pure, unconfounded indicator of reduced ventricular wall stress in this titrated cohort. In the HFmrEF subgroup, the lower overall adoption of ARNI use in real-world registries during the study era minimized this confounding effect on BNP, whereas the lack of statistical significance in NT-proBNP was highly likely due to a type II error stemming from the small sample size of longitudinal measurements. Collectively, these multidimensional insights underscore that a higher combination dose of contemporary quadruple therapy standardly facilitates structural and biochemical recovery, irrespective of the baseline LVEF phenotype.

HF in contemporary practice is increasingly encountered in older patients with compromised nutritional status and frailty—factors known to independently affect outcomes.^36^, ^37^, ^38^, ^39^, ^40^ In our study, the prognostic advantage of a higher simple GDMT score remained consistent regardless of baseline nutritional status (as measured by GNRI) or frailty (as measured by the Clinical Frailty Scale), suggesting that even vulnerable populations may benefit from aggressive and optimized GDMT. Given Japan's aging population, the simple GDMT score may serve as a practical tool for discharge planning and treatment optimization in routine clinical care. Despite the robust evidence supporting GDMT, its implementation remains suboptimal both in Japan and globally. This so-called "evidence–practice gap" is a major challenge in HF management.^18^, ^19^, ^20^, ^21^^,^^41^ The simple GDMT score, by offering a straightforward and quantifiable measure of therapy implementation, may serve as a useful clinical tool to improve GDMT adoption. In the future, this score could be incorporated into interventional trials or regional healthcare coordination frameworks to assess whether its use leads to improved implementation rates and patient outcomes.

Recently, a structured optimal medical therapy score was reported, and a higher score was significantly correlated with a better out-of-hospital prognosis, strongly reinforcing the validity of our findings.^42^ However, a critical distinction between the scores, and a unique benefit of our simple GDMT score over the previously reported optimal medical therapy score is the explicit incorporation of SGLT2 inhibitors. Although historical scores often omit SGLT2 inhibitors because they were derived from older patient cohorts, contemporary international guidelines now strongly mandate standard quadruple therapy ("the fantastic four") as the frontline regimen for HF. By integrating SGLT2 inhibitors along with detailed dose-optimization metrics for RAS inhibitors, beta-blockers, and MRAs, our score provides a more comprehensive, updated, and practical benchmark that accurately reflects the clinical realities of the modern quadruple therapy era. Consequently, our simple GDMT score may serve as a more useful and contemporaneous bedside tool to bridge the evidence–practice gap in daily routine care.

This study has several limitations. First, due to the limited observation period, we were unable to track long-term adherence or changes in medication regimens beyond discharge. Second, survival bias may have influenced the estimation of changes in echocardiographic and biomarker parameters, as only patients who survived to the 1-year follow-up assessment were included in those analyses. Third, during the study period, the prescription rates of newer agents such as ARNIs and SGLT2 inhibitors remained relatively low, and their use may have varied across institutions, potentially introducing centre-level bias. Fourth, this study did not utilize an independent external validation cohort, which limits the immediate generalizability of the prognostic associations observed. Although the consistency and certainty of the score's independent association were rigorously substantiated via multi-layered sensitivity analyses, adjusting for frailty, nutrition, and independent age components, external replication in distinct geographic or institutional cohorts remains necessary to establish the universal clinical utility of the score. aAnother major limitation is the lack of detailed information regarding medication use and dosage adjustment during the follow-up period. Because GDMT optimization is a dynamic process that continues after hospital discharge, any subsequent changes in therapy—such as dose titration, the initiation of additional “pillars,” or the discontinuation of medications—could have significantly influenced cardiac reverse remodelling and clinical outcomes. Therefore, our results should be interpreted as reflecting the prognostic impact of the treatment status at the time of discharge, rather than the entire course of medical management. In addition, the ability to tolerate higher doses of GDMT may simply reflect a patient’s inherent clinical robustness or physiological reserve rather than the direct therapeutic effect of the medications themselves. Although we cannot completely exclude this selection bias, our multivariate analysis demonstrated that the GDMT score remained a strong independent predictor of outcomes even after adjusting for age and renal function. Additionally, the score's consistent performance in the subgroup analysis of patients with high CFS scores suggests that its prognostic value extends beyond baseline physical robustness. Nevertheless, further prospective studies are needed to confirm the causal relationship between GDMT optimization based on this score and improved clinical outcomes. Future studies in more diverse and contemporary populations are warranted to validate these findings.

Looking forward, the simple GDMT score may also provide a framework for interventional trials aimed at improving GDMT implementation. Embedding this score into clinical workflows and using it to trigger targeted interventions (eg, pharmacist-led optimization, multidisciplinary care) could determine whether active use of the score improves implementation rates and patient outcomes. Additionally, global validation of the score in diverse healthcare systems is essential before its widespread adoption.

## Conclusion

The simple GDMT score at discharge provides a practical, straightforward, clinical benchmark to quantify the implementation intensity of foundational HF therapies in real-world practice. A higher score, reflecting more optimized combination therapy at discharge, was significantly associated with favourable 2-year clinical outcomes and more pronounced longitudinal cardiac reverse remodelling across both the HFrEF and HFmrEF phenotypes within this specific registry cohort. Rather than operating as a standalone high-accuracy risk-prediction model, this simple tool may serve as an indicator for discharge planning and treatment optimization to ensure GDMT implementation in routine clinical care.

## Acknowledgements

This study was conducted on behalf of all the JROADHF-NEXT investigators. JROADHF-NEXT was supported by the Japanese Circulation Society and the Japanese Society of Heart Failure.

## Data Availability

The deidentified participant data are shared with only the JROADHF-NEXT investigators.

## Ethics Statement

This study was conducted in accordance with the Declaration of Helsinki. The study protocol was approved by the institutional review boards of Kyushu University (No. 2019-023), the International University of Health and Welfare (No. 24-KS-005), and all participating institutions.

## Patient Consent

The authors confirm that a patient consent form has been obtained for this article.

## Funding Sources

This work was supported by a 10.13039/100009619Japan Agency for Medical Research and Development grant (19ek0210080h0003 [H.T.]) and Health Sciences Research Grants from the Japanese 10.13039/100009647Ministry of Health, Labour and Welfare (Comprehensive Research on Cardiovascular Diseases; 20FC1051 [H.T.]). The funders had no role in the design or conduct of the study or in the collection, management, analysis, or interpretation of the data, or in the preparation, review, and submission of the article for publication.

## Disclosures

R.M. reports receiving lecture fees from Otsuka Pharmaceutical, AstraZeneca, Novartis Japan, Ono Pharmaceutical, and Boehringer Ingelheim. Y. Mukai reports receiving lecture fees from Daiichi Sankyo and Medtronic. Y. Matsue reports receiving honoraria from Otsuka Pharmaceutical Co., EN Otsuka Pharmaceutical Co., Novartis Pharma K.K., Bayer Inc., Pfizer Japan Inc., Nippon Boehringer Ingelheim Co., and AstraZeneca, and a collaborative research grant from Pfizer Japan Inc., Otsuka Pharmaceutical Co., EN Otsuka Pharmaceutical Co., Ltd., Roch Diagnostics K.K., and Nippon Boehringer Ingelheim Co. T.S. reports receiving lecture fees from Astellas Pharma Inc., Ono Pharmaceutical Co., Ltd., Otsuka Pharmaceutical Co., Boehringer Ingelheim, Novartis Pharma K.K., Bristol-Myers Squibb Company, Daiichi Sankyo Co., Ltd., Bayer Inc., Medtronic, Abbott, and Edwards Lifesciences Corporation. N.E. reports receiving support from an American Heart Association Postdoctoral Fellowship, the Uehara Memorial Foundation Fellowship, and the Tsuchiya Memorial Foundation Fellowship.T.T. reports being affiliated with the collaborative research division of Mochida Pharmaceuticals (FY2024; April 1, 2023 – March 31, 2024) and being supported by the Cardiovascular Innovative Conference grant and the Uehara Memorial Foundation Fellowship. S.M. reports receiving grants from Tsumura & Co. and Astellas Pharma Inc. T.K. reports receiving lecture fees from Alnylam Japan, AstraZeneca K.K., Otsuka Pharmaceutical Co., Ltd., Ono Pharmaceutical Co., Ltd., Kowa Co., Ltd., Eli Lilly Japan K.K., Nippon Boehringer Ingelheim Co., Ltd., Novartis Pharma K.K., Novo Nordisk Pharma Ltd., Bayer Yakuhin, Ltd., and Pfizer Japan Inc.; and receiving research funding or joint research expenses from Kowa Co., Ltd.K.K. reports receiving honoraria from AstraZeneca K.K., Ono Pharmaceutical Co., Ltd., Nippon Boehringer Ingelheim Co., Ltd., Bayer Yakuhin, Ltd., Otsuka Pharmaceutical Co., Ltd., and Novartis Pharmaceuticals Co., Ltd. T.O. reports being affiliated with a department sponsored by Medtronic Japan, receiving research grants from Pfizer Japan, Alnylam Japan Pharmaceuticals, and Alexion Pharmaceuticals, and lecture fees from Pfizer Japan, Novartis Pharma, AstraZeneca, and Boehringer Ingelheim, not connected to this work. T.T. reports being the founder and a shareholder of Remohab Inc. H.T. reports being a consultant to Nippon Boehringer Ingelheim Co., Ltd., Bayer Yakuhin, Ltd., Novartis Pharma K.K., and Ono Pharmaceutical Co., Ltd.; receiving remuneration from MSD K.K., Astellas Pharma Inc., Pfizer Japan Inc., Bristol-Myers Squibb Company, Otsuka Pharmaceutical Co., Ltd., Daiichi Sankyo Co., Ltd., Mitsubishi Tanabe Pharma Corporation, Nippon Boehringer Ingelheim Co., Ltd., Takeda Pharmaceutical Company Limited, Bayer Yakuhin, Ltd., Novartis Pharma K.K., Kowa Pharmaceutical Co. Ltd., and Teijin Pharma Ltd.; manuscript fees from Medical View, Nippon Rinsho; research funding from Actelion Pharmaceuticals Japan Ltd., Mitsubishi Tanabe Pharma Corporation, Nippon Boehringer Ingelheim Co., Ltd., Daiichi Sankyo Co., Ltd., IQVIA Services Japan, Omron Healthcare; and scholarship funds from Astellas Pharma Inc., Novartis Pharma K.K., Daiichi Sankyo Co., Ltd., Takeda Pharmaceutical Company Limited, Mitsubishi Tanabe Pharma Corporation, Teijin Pharma Ltd., and MSD K.K. K.A. reports receiving a grant from Konica Minolta and Daiichi Sankyo outside the submitted work.
