# Supplementary material for: Prognostic Impact of a Simple GDMT Score on Clinical Outcomes and Cardiac Reverse Remodelling in Patients with ADHF: Insights from the JROADHF-Next Registry
Source: CJC Open. 2026 Jul 20;8(10):1310–23. doi: 10.1016/j.cjco.2026.06.022 (PMC13654385; doi:10.1016/j.cjco.2026.06.022)

**Supplemental Table S1. Drug dosages were assessed relative to the maximum approved doses in Japan**

| | Max dose |
|---|---|
| ACE inhibitor | |
| Enalapril | 10mg |
| Lisinopril | 10mg |
| Imidapril | 10mg |
| Perindopril | 8mg |
| Captopril | 150mg |
| Alacepril | 100mg |
| Cilazapril | 2mg |
| trandolapril | 2mg |
| Temocapril | 4mg |
| ARB | |
| Candesartan | 12mg |
| Azilsartan | 40mg |
| Olmesartan | 40mg |
| Irubesartan | 200mg |
| Losartan | 100mg |
| Terumisartan | 80mg |
| Valsartan | 160mg |
| Beta-blocker | |
| Carvedilol | 20mg |
| Bisoprolol | 5mg |

**Supplemental Table S2. Characteristics and medication at discharge between Simple GDMT score low and high group (HFrEF)**

| HFrEF (n=1729) | | | |
|---|---|---|---|
| | Simple GDMT score Low (n=908) | Simple GDMT score High (n=821) | p-value |
| **Backgrounds** | | | |
| Age (years) | 70.6±14.3 | 64.2±14.1 | <0.001 |
| Male | 618 (68.1) | 627 (76.4) | <0.001 |
| Prior HF hospitalization | 381 (42.0) | 309 (37.6) | 0.060 |
| NYHA 3-4 at admission (%) | 880 (88.1) | 739 (90.0) | 0.218 |
| **Etiology** | | | |
| IHD (%) | 252 (27.8) | 216 (26.3) | 0.516 |
| Cardiomyopathy (%) | 322 (35.5) | 340 (41.4) | 0.012 |
| Hypertensive heart disease (%) | 93 (10.2) | 119 (14.5) | 0.008 |
| Valvular heart disease (%) | 97 (10.7) | 35 (4.3) | <0.001 |
| Others (%) | 144 (15.9) | 111 (13.5) | 0.175 |
| **Comorbidities** | | | |
| AF (%) | 316 (34.8) | 259 (31.5) | 0.153 |
| DM (%) | 266 (29.3) | 365 (44.5) | <0.001 |
| DL (%) | 493 (38.6) | 435 (41.9) | 0.115 |
| COPD (%) | 62 (6.8) | 48 (5.8) | 0.431 |
| SAS (%) | 39 (4.3) | 83 (10.1) | <0.001 |
| Cancer | 84 (9.3) | 57 (6.9) | 0.094 |
| **Characteristics at discharge** | | | |
| NYHA 3-4 (%) | 133 (14.7) | 78 (9.5) | 0.001 |
| Systolic BP (mmHg) | 106.9±17.0 | 106.2±16.7 | 0.390 |
| Diastolic BP (mmHg) | 63 (56, 70) | 64 (57, 73) | 0.002 |
| Heart rate (bpm) | 70 (63, 80) | 70 (63, 78) | 0.343 |
| BMI (kg/m2) | 20.9 (18.6, 23.2) | 22.5 (20.0, 25.4) | <0.001 |
| GNRI | 90.8±9.0 | 94.7±8.0 | <0.001 |
| Clinical frailty scale | 4 (3, 5) | 3 (3, 4) | <0.001 |
| EF at discharge (%) | 29.3±7.3 | 28.4±6.9 | 0.010 |
| **Laboratory data at discharge** | | | |
| BNP (pg/ml) | 493.0±560.1 | 378.5±423.9 | <0.001 |
| NT-proBNP (pg/ml) | 2176.5 (980.7, 4818.2) | 1468.5 (662.2, 2912.0) | <0.001 |

| | | | |
|---|---|---|---|
| Hemoglobin (g/dl) | 12.5±2.3 | 13.5±2.3 | <0.001 |
| BUN (mg/dl) | 24.0 (18.0, 36.0) | 22.0 (17.0, 30.0) | <0.001 |
| Creatinine (mg/dl) | 1.15 (0.88 1.60) | 1.10 (0.89, 1.42) | 0.019 |
| eGFR (ml/min/1.73m2) | 45.2 (31.5, 60.4) | 49.7 (38.0, 62.8) | <0.001 |
| Sodium (mEq/L) | 139.0 (136.0, 141.0) | 139.0 (137.0, 141.0) | 0.332 |
| Pottasium (mEq/L) | 4.3 (4.0, 4.7) | 4.4 (4.1, 4.7) | 0.332 |
| Chloride (mEq/L) | 102.0 (100.0, 105.0) | 102 (100.0, 105.0) | 0.698 |
| Albumin (mg/dl) | 3.5±0.4 | 3.7±0.4 | <0.001 |
| UA (mg/dl) | 7.0±2.1 | 6.7.5±1.8 | 0.005 |
| **Medication at discharge** | | | |
| Hospital stay (days) | 27.9±24.3 | 25.2±22.6 | 0.025 |
| Simple GDMT score (pts) | 2.9±1.1 | 5.9±1.0 | <0.001 |
| RAS inhibitor (%) | 631 (78.9) | 788 (97.6) | <0.001 |
| ARNI (%) | 8 (1.4) | 67 (12.2) | <0.001 |
| Beta-blocker (%) | 775 (87.4) | 808 (98.4) | <0.001 |
| MRA (%) | 463 (51.0) | 759 (92.4) | <0.001 |
| SGLT2 inhibitor (%) | 64 (7.0) | 372 (45.3) | <0.001 |
| Ivabradine (%) | 19 (3.4) | 20 (3.6) | 0.872 |
| Loop diuretics (%) | 763 (84.0) | 700 (85.3) | 0.505 |
| Loop diuretics dose (mg) | 20.0 (10.0, 40.0) | 20.0 (10.0, 40.0) | 0.217 |
| TLV (%) | 333 (36.7) | 277 (33.7) | 0.208 |
| Pimobendan (%) | 161 (17.7) | 105 (12.8) | 0,005 |

**Supplemental Table S3. Characteristics and medication at discharge between Simple GDMT score low and high group (HFmrEF)**

| HFmrEF (n=584) | | | |
|---|---|---|---|
| | Simple GDMT score Low (n=386) | Simple GDMT score High (n=216) | p-value |
| **Backgrounds** | | | |
| Age (years) | 76.2±12.5 | 71.9±12.5 | <0.001 |
| Male | 214 (58.2) | 147 (68.1) | 0.018 |
| Prior HF hospitalization | 123 (33.4) | 69 (31.9) | 0.784 |
| NYHA 3-4 at admission (%) | 327 (88.9) | 196 (90.7) | 0.575 |
| **Etiology** | | | |
| IHD (%) | 101 (27.4) | 73 (33.8) | 0,112 |
| Cardiomyopathy (%) | 66 (17.9) | 40 (18.5) | 0.912 |
| Hypertensive heart disease (%) | 55 (14.9) | 27 (12.5) | 0.460 |
| Valvular heart disease (%) | 65 (17.7) | 28 (13.0) | 0.160 |
| Others (%) | 81 (22.0) | 48 (22.2) | 1.000 |
| **Comorbidities** | | | |
| AF (%) | 172 (46.7) | 91 (42.1) | 0.302 |
| DM (%) | 113 (30.7) | 124 (57.4) | <0.001 |
| DL (%) | 143 (38.9) | 95 (44.0) | 0.257 |
| COPD (%) | 24 (6.5) | 11 (5.1) | 0.589 |
| SAS (%) | 18 (4.9) | 8 (3.7) | 0.542 |
| Cancer | 31 (8.4) | 18 (8.3) | 1.000 |
| **Characteristics at discharge** | | | |
| NYHA 3-4 (%) | 38 (10.4) | 9 (4.2) | 0.007 |
| Systolic BP (mmHg) | 115.0±16.9 | 113.5±16.8 | 0.305 |
| Diastolic BP (mmHg) | 64 (57, 72) | 65 (56, 72) | 0.665 |
| Heart rate (bpm) | 70 (63, 79) | 70 (61, 78) | 0.304 |
| BMI (kg/m2) | 21.2 (18.8, 23.5) | 22.2 (19.9, 25.1) | 0.001 |
| GNRI | 89.9±8.8 | 91.5±8.9 | 0.043 |
| Clinical frailty scale | 4 (3, 5) | 4 (3, 5) | 0.001 |
| EF at discharge (%) | 44.8±2.4 | 44.8±2.6 | 0.980 |
| **Laboratory data at discharge** | | | |
| BNP (pg/ml) | 388.2±450.8 | 323.6±307.1 | 0.123 |
| NT-proBNP (pg/ml) | 2094.0 (972.0, 5090.0) | 1096.5 (658.9, 2429.5) | 0.001 |

| | | | |
|---|---|---|---|
| Hemoglobin (g/dl) | 11.7±2.3 | 12.4±2.4 | <0.001 |
| BUN (mg/dl) | 27.0 (19.1, 37.0) | 22.9 (18.3, 29.8) | 0.001 |
| Creatinine (mg/dl) | 1.23 (0.92, 1.76) | 1.12 (0.91, 1.39) | 0.020 |
| eGFR (ml/min/1.73m2) | 40.4 (27.1, 55.9) | 45.6 (36.9, 57.2) | 0.001 |
| Sodium (mEq/L) | 139.0 (137.0, 141.0) | 139.0 (137.0, 141.0) | 0.104 |
| Pottasium (mEq/L) | 4.3 (4.0, 4.6) | 4.3 (4.1, 4.6) | 0.693 |
| Chloride (mEq/L) | 102.0 (99.0, 105.0) | 103 (101.0, 105.0) | 0.079 |
| Albumin (mg/dl) | 3.4±0.4 | 3.5±0.5 | 0.247 |
| UA (mg/dl) | 7.1±2.2 | 6.7±1.9 | 0.026 |
| **Medication at discharge** | | | |
| Hospital stay (days) | 24.5±18.9 | 21.5±15.3 | 0.061 |
| Simple GDMT score (pts) | 2.5±1.2 | 5.8±0.9 | <0.001 |
| RAS inhibitor (%) | 238 (75.8) | 202 (94.8) | <0.001 |
| ARNI (%) | 1 (0.4) | 12 (8.3) | <0.001 |
| Beta-blocker (%) | 277 (75.3) | 211 (97.7) | <0.001 |
| MRA (%) | 141 (38.3) | 195 (53.2)195 (90.3) | <0.001 |
| SGLT2 inhibitor (%) | 24 (6.5) | 102 (47.2) | <0.001 |
| Ivabradine (%) | 1 (0.4) | 2 (1.4) | 0.559 |
| Loop diuretics (%) | 296 (80.4) | 181 (83.8) | 0.321 |
| Loop diuretics dose (mg) | 20.0 (10.0, 40.0) | 20.0 (10.0, 30.0) | 0.229 |
| TLV (%) | 103 (28.0) | 63 (29.2) | 0.321 |
| Pimobendan (%) | 16 (4.3) | 11 (5.1) | 0.687 |

**Supplemental Table S4. Multivariable Cox proportional hazards analysis (HFrEF/HFmrEF)**

| | Unadjusted model | | | Adjusted model | | |
|---|---|---|---|---|---|---|
| | HR | 95%CI | p-value | HR | 95%CI | p-value |
| Composite outcome (HFrEF) | 0.67 | 0.55-0.81 | <0.001 | 0.73 | 0.57-0.93 | 0.01 |
| Composite outcome (HFmrEF) | 0.58 | 0.42-0.81 | <0.001 | 0.54 | 0.31-0.94 | 0.03 |

## Supplemental Table S5. Sensitivity Analysis

| Model | Adjustment Covariates | HR | 95% CI | p-value | Analyzed N | Events |
|---|---|---|---|---|---|---|
| Adjusted Model (Baseline Model) | GWTG-HF risk score, eGFR, log-transformed BNP at discharge, NYHA class at discharge, LVEF at discharge, anemia, and prior HF hospitalization | 0.708 | 0.569–0.881 | 0.002 | 1246 | 346 |
| Model A (Vulnerability Adjusted) | Model 1 covariates + Geriatric Nutritional Risk Index (GNRI) + Clinical Frailty Scale (CFS) | 0.760 | 0.605–0.955 | 0.018 | 1177 | 325 |
| Model B (Dismantled Age & Vulnerability) | Age, GNRI, CFS, eGFR, log-transformed BNP at discharge, NYHA class at discharge, LVEF at discharge, anemia, and prior HF hospitalization | 0.806 | 0.656–0.989 | 0.039 | 1492 | 411 |

**Supplemental Table S6. Changes in echocardiographic and biomarker parameters between discharge and 12 months (HFrEF)**

| Parameter | Group | At Discharge | p-value | At 1-Year Follow-up | p-value | Absolute Change (Δ) | p-value | % Change from Baseline | p-value |
|---|---|---|---|---|---|---|---|---|---|
| LVEF (%) | Low Score (n=869) | 30.0 (24.0–35.0) [n=869] | 0.003 | 40.0 (30.0–51.2) [n=588] | 0.296 | 10.0 (1.0–21.0) [n=588] | 0.012 | 34.6 (3.8–75.9) [n=588] | 0.014 |
| | High Score (n=791) | 28.0 (23.0–33.0) [n=791] | | 42.0 (30.0–54.0) [n=621] | | 13.0 (3.0–25.0) [n=621] | | 45.7 (10.7–84.8) [n=621] | |
| LVEDVI (mL/m²) | Low Score (n=869) | 107.4 (87.7–129.5) [n=866] | 0.052 | 85.5 (63.5–113.2) [n=534] | 0.585 | -16.2 (-36.1–-0.2) [n=534] | 0.008 | -14.7 (-33.8–-0.2) [n=534] | 0.012 |
| | High Score (n=791) | 109.1 (90.0–135.4) [n=789] | | 83.3 (61.2–116.8) [n=588] | | -21.5 (-41.5–-3.5) [n=586] | | -19.9 (-38.2–-3.0) [n=586] | |
| LVESVI (mL/m²) | Low Score (n=869) | 75.3 (57.4–95.7) [n=865] | 0.006 | 48.9 (30.1–78.7) [n=534] | 0.473 | -20.1 (-38.4–-2.4) [n=533] | 0.003 | -27.4 (-54.4–-2.5) [n=533] | 0.005 |
| | High Score (n=791) | 77.8 (61.3–102.7) [n=788] | | 45.7 (26.8–84.1) [n=588] | | -25.0 (-43.7–-6.7) [n=586] | | -33.2 (-60.2–-7.6) [n=586] | |
| LAVI (mL/m²) | Low Score (n=869) | 50.3 (38.5–68.8) [n=556] | 0.011 | 38.8 (26.7–56.8) [n=366] | 0.086 | -7.8 (-19.9–3.2) [n=305] | 0.244 | -16.2 (-39.1–7.4) [n=305] | 0.307 |
| | High Score (n=791) | 48.5 (36.7–62.7) [n=521] | | 35.6 (25.5–54.2) [n=424] | | -9.6 (-20.0–0.6) [n=352] | | -19.9 (-38.8–1.2) [n=352] | |
| LVMI (g/m²) | Low Score (n=869) | 136.9 (110.6–162.3) [n=861] | 0.658 | 115.9 (91.4–143.3) [n=531] | 0.047 | -14.7 (-38.6–3.9) [n=528] | 0.012 | -11.5 (-27.9–2.9) [n=528] | 0.013 |
| | High Score (n=791) | 136.3 (113.9–160.8) [n=789] | | 110.2 (90.7–137.9) [n=584] | | -22.3 (-43.6–-0.0) [n=582] | | -16.5 (-30.5–-0.0) [n=582] | |
| E/E' | Low Score (n=869) | 16.7 (11.3–23.4) [n=729] | 0.801 | 12.6 (9.4–19.1) [n=479] | 0.004 | -2.2 (-7.5–1.9) [n=433] | 0.036 | -15.3 (-38.6–14.2) [n=433] | 0.011 |
| | High Score (n=791) | 16.3 (11.9–23.0) [n=648] | | 11.5 (8.6–16.6) [n=514] | | -3.2 (-8.4–0.9) [n=450] | | -22.9 (-45.6–7.6) [n=450] | |
| BNP (pg/mL) | Low Score (n=869) | 338.9 (158.8–615.0) [n=580] | <0.001 | 152.0 (37.1–415.1) [n=432] | 0.003 | -0.6 (-1.7–0.1) [n=407] | 0.082 | -11.0 (-31.9–2.6) [n=407] | 0.077 |
| | High Score (n=791) | 277.9 (132.3–484.2) [n=568] | | 106.5 (29.7–292.6) [n=459] | | -0.8 (-1.9–0.1) [n=448] | | -15.7 (-34.7–1.3) [n=448] | |
| NT-proBNP (pg/mL) | Low Score (n=869) | 2176.5 (984.2–4747.0) [n=296] | <0.001 | 1305.0 (295.0–4001.0) [n=265] | <0.001 | -0.6 ± 1.5 [n=219] | <0.001 | -7.6 (-21.9–4.4) [n=219] | <0.001 |
| | High Score (n=791) | 1510.5 (689.2–2967.5) [n=262] | | 555.0 (160.0–1885.0) [n=279] | | -1.1 ± 1.4 [n=219] | | -14.4 (-29.6–-2.4) [n=219] | |
| eGFR (mL/min/1.73m²) | Low Score (n=869) | 45.3 (31.8–60.4) [n=867] | <0.001 | 41.8 (29.8–59.3) [n=709] | <0.001 | -1.6 (-9.8–6.4) [n=709] | 0.865 | -4.7 (-22.5–16.0) [n=709] | 0.511 |
| | High Score (n=791) | 49.9 (38.1–62.8) [n=791] | | 48.0 (35.0–62.8) [n=718] | | -1.5 (-9.8–7.3) [n=717] | | -3.0 (-19.6–16.0) [n=717] | |

## Supplemental Table S7. Changes in echocardiographic and biomarker parameters between discharge and 12 months (HFmrEF)

| Parameter | Group | At Discharge | p-value | At 1-Year Follow-up | p-value | Absolute Change (Δ) | p-value | % Change from Baseline | p-value |
|---|---|---|---|---|---|---|---|---|---|
| LVEF (%) | Low Score (n=407) | 44.0 (42.0–47.0) [n=407] | 0.549 | 50.0 (41.5–58.0) [n=263] | 0.021 | 5.2 ± 11.1 [n=263] | 0.014 | 11.8 ± 25.3 [n=263] | 0.013 |
| | High Score (n=246) | 44.0 (42.0–47.0) [n=246] | | 53.0 (45.0–62.0) [n=170] | | 7.9 ± 11.2 [n=170] | | 18.1 ± 26.1 [n=170] | |
| LVEDVI (mL/m²) | Low Score (n=407) | 79.6 (64.9–99.3) [n=404] | 0.919 | 71.0 (58.0–90.0) [n=240] | 0.292 | -4.1 (-19.1–4.8) [n=240] | 0.011 | -5.9 (-23.2–6.1) [n=240] | 0.014 |
| | High Score (n=246) | 81.3 (67.3–96.8) [n=246] | | 68.9 (57.9–84.3) [n=160] | | -11.1 (-24.6–1.6) [n=160] | | -14.4 (-27.3–2.4) [n=160] | |
| LVESVI (mL/m²) | Low Score (n=407) | 43.9 (34.4–56.9) [n=402] | 0.746 | 31.4 (23.4–51.2) [n=240] | 0.355 | -6.8 (-19.5–2.8) [n=239] | 0.027 | -16.5 (-41.9–5.5) [n=239] | 0.034 |
| | High Score (n=246) | 44.0 (35.6–55.4) [n=246] | | 31.1 (22.4–43.4) [n=160] | | -11.4 (-21.9–0.1) [n=160] | | -28.5 (-46.1–0.1) [n=160] | |
| LAVI (mL/m²) | Low Score (n=407) | 47.6 (37.6–64.9) [n=281] | 0.041 | 42.6 (30.2–60.7) [n=178] | 0.653 | -4.3 (-14.4–6.8) [n=156] | 0.784 | -8.1 (-30.3–20.0) [n=156] | 0.990 |
| | High Score (n=246) | 46.2 (34.2–55.4) [n=175] | | 42.6 (29.4–59.2) [n=121] | | -3.0 (-12.0–5.5) [n=103] | | -7.5 (-25.9–10.7) [n=103] | |
| LVMI (g/m²) | Low Score (n=407) | 122.5 (102.4–149.8) [n=403] | 0.950 | 110.9 (91.6–140.0) [n=240] | 0.193 | -9.0 (-29.1–8.0) [n=239] | 0.191 | -6.9 (-22.6–6.8) [n=239] | 0.110 |
| | High Score (n=246) | 124.2 (106.7–147.0) [n=246] | | 112.1 (88.2–130.3) [n=159] | | -14.6 (-31.1–1.9) [n=159] | | -11.7 (-24.4–1.8) [n=159] | |
| E/E' | Low Score (n=407) | 16.4 (11.6–22.3) [n=350] | 0.162 | 14.4 (10.0–19.8) [n=225] | 0.829 | -0.7 (-6.1–4.0) [n=206] | 0.841 | -4.7 (-33.3–29.8) [n=206] | 0.631 |
| | High Score (n=246) | 15.7 (11.4–21.0) [n=213] | | 13.5 (10.0–19.3) [n=138] | | -1.5 (-5.0–2.8) [n=131] | | -9.5 (-32.0–20.3) [n=131] | |
| BNP (pg/mL) | Low Score (n=407) | 242.4 (109.4–501.6) [n=259] | 0.260 | 187.7 (77.3–412.3) [n=185] | 0.009 | -0.2 (-0.9–0.5) [n=172] | 0.013 | -3.0 (-15.8–9.1) [n=172] | 0.018 |
| | High Score (n=246) | 213.8 (112.5–410.2) [n=172] | | 122.2 (56.6–292.4) [n=136] | | -0.4 (-1.2–0.2) [n=127] | | -7.7 (-23.9–3.0) [n=127] | |
| NT-proBNP (pg/mL) | Low Score (n=407) | 2094.0 (961.0–5289.0) [n=141] | <0.001 | 1553.0 (499.8–5158.0) [n=120] | <0.001 | -0.2 ± 1.4 [n=103] | 0.211 | -3.1 ± 19.1 [n=103] | 0.163 |

| | | | | | | | | | |
|---|---|---|---|---|---|---|---|---|---|
| | High Score (n=246) | 1068.5 (630.8–2238.0) [n=88] | | 901.0 (293.8–1564.2) [n=82] | | -0.5 ± 1.2 [n=65] | | -7.2 ± 17.5 [n=65] | |
| **eGFR (mL/min/1.73m²)** | Low Score (n=407) | 40.4 (27.0–56.2) [n=406] | <0.001 | 39.8 (26.0–53.7) [n=322] | 0.002 | -3.1 (-11.0–2.5) [n=322] | 0.044 | -8.3 (-25.2–8.9) [n=322] | 0.185 |
| | High Score (n=246) | 45.7 (37.0–57.1) [n=246] | | 44.4 (32.9–55.8) [n=213] | | -5.2 (-15.0–1.9) [n=213] | | -11.5 (-26.6–4.1) [n=213] | |

## Supplemental Figure S1 ROC curve

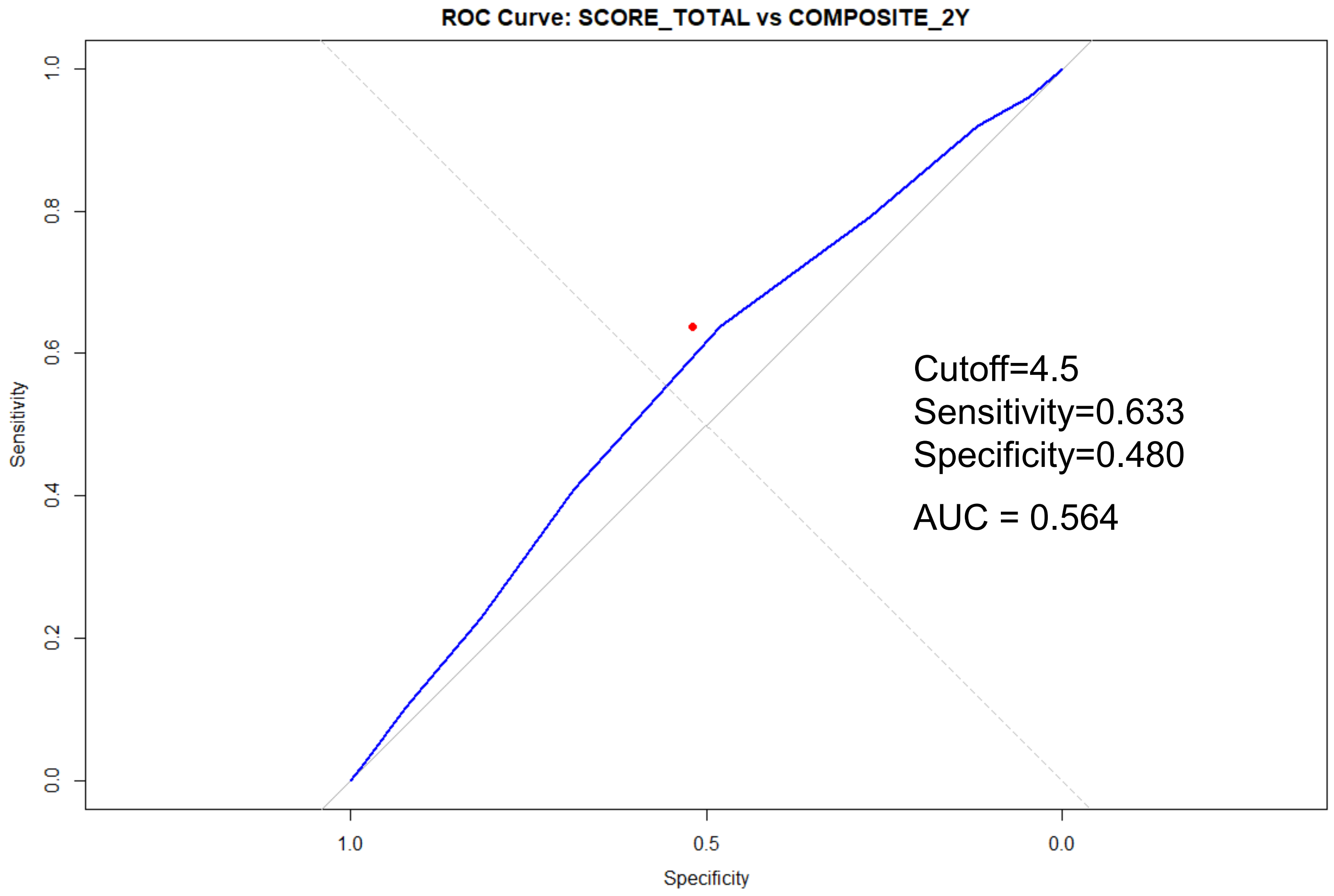

**Supplemental Figure S2 Distribution of simple GDMT score**

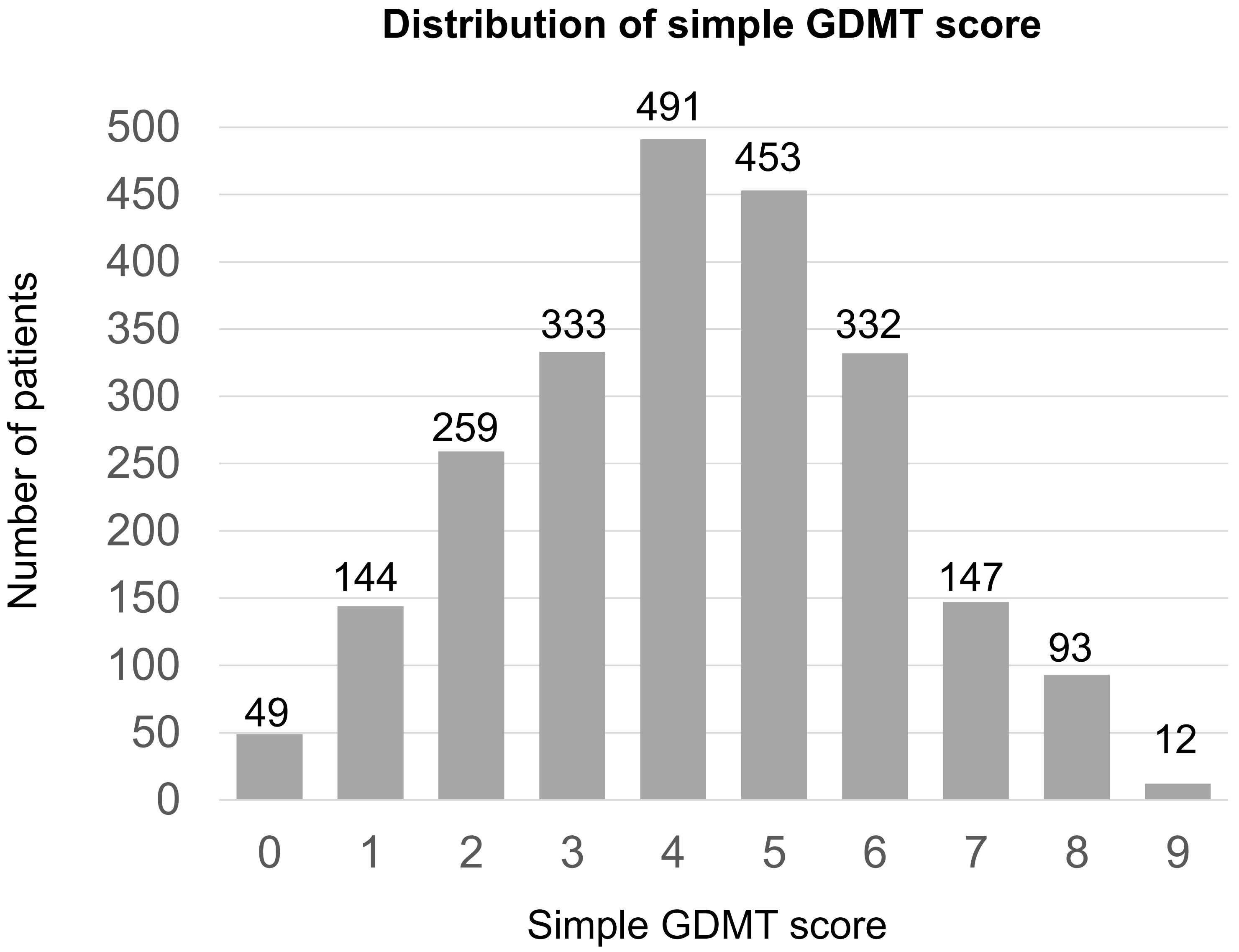

## Supplemental Figure S3 2-year prognosis in HFrEF and HFmrEF

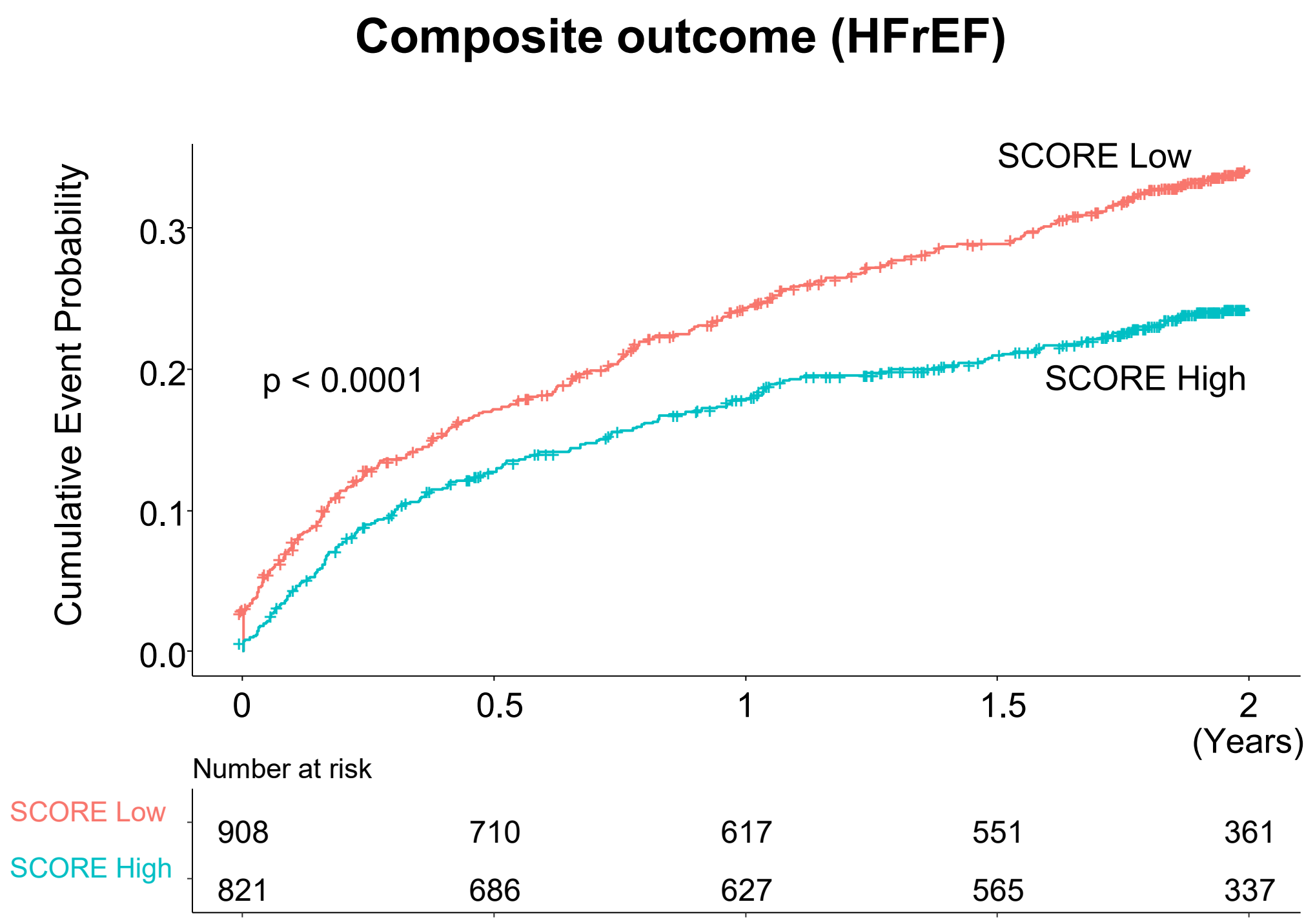


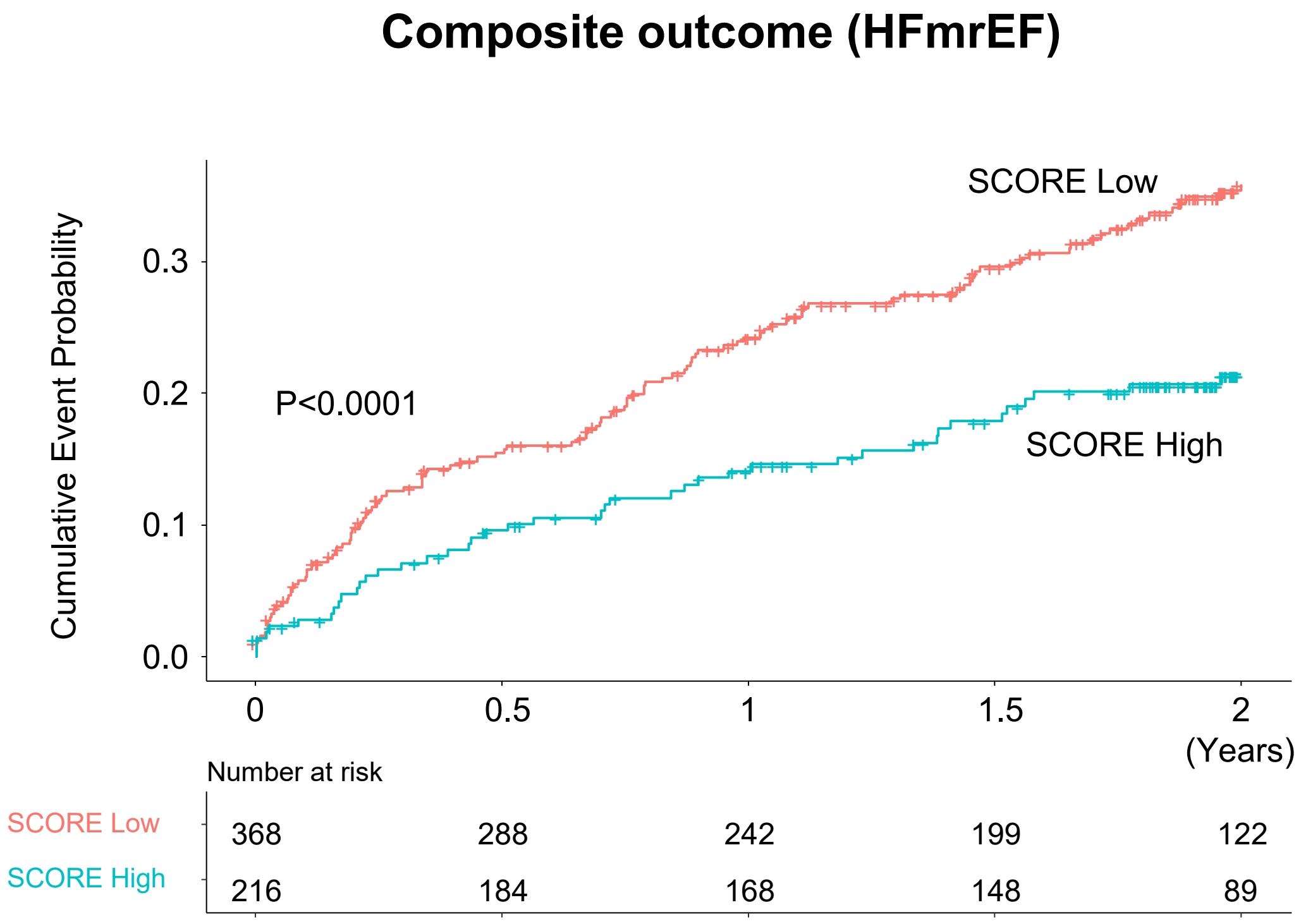

Supplemental Figure S4A %Changes in echo parameters (HFrEF)

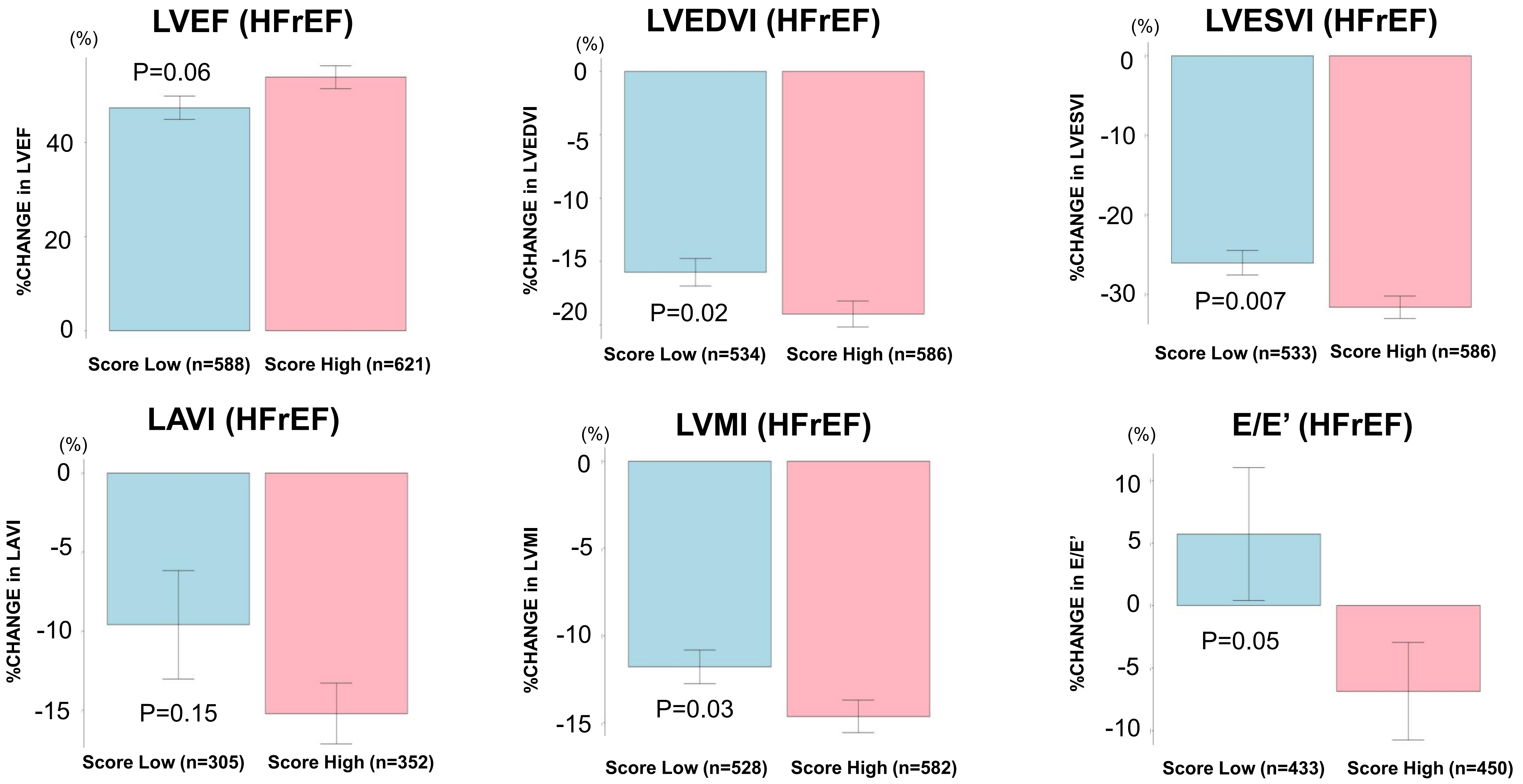

## Supplemental Figure S5 %Changes in echo parameters (HFmrEF)

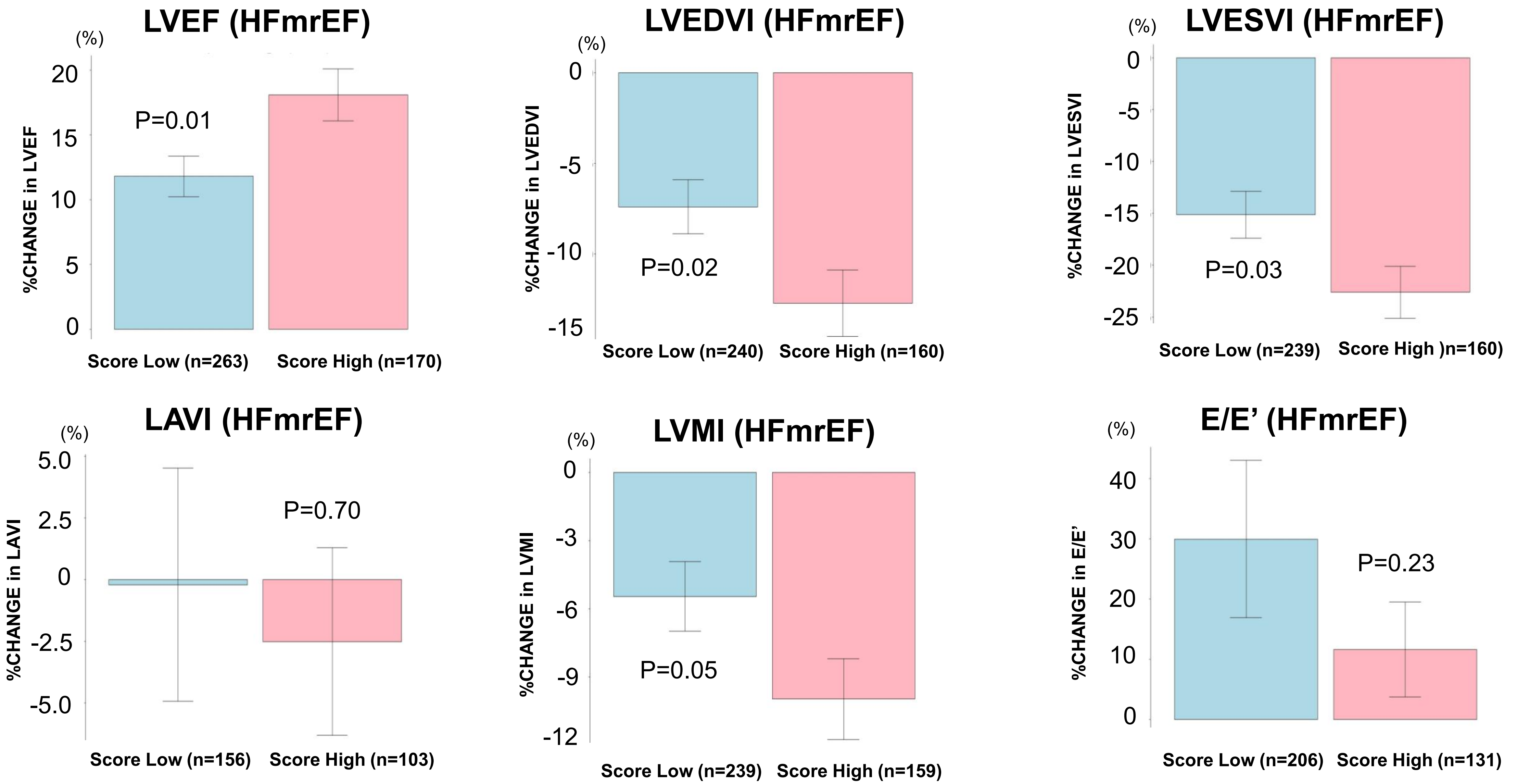

## Supplemental Figure S6 %Changes in biomarkers (HFrEF and HFmrEF)

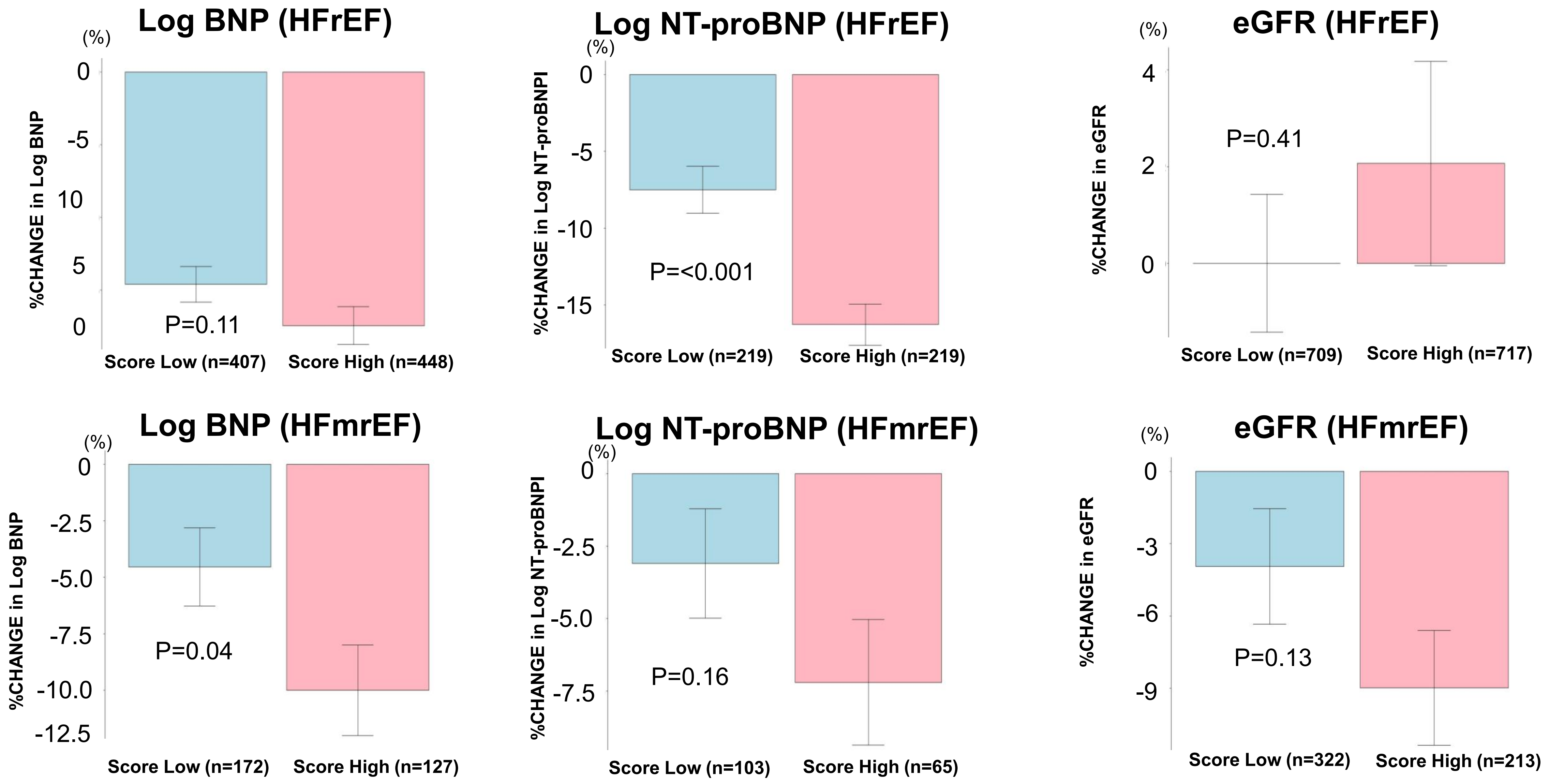

Supplement: Supplementary file 1 [file mmc1.pdf]
